# Effect of high-dose dexamethasone on morphine use after periacetabular osteotomy for hip dysplasia: a randomized double-blind placebo-controlled single center trial

**DOI:** 10.2340/17453674.2025.43903

**Published:** 2025-06-01

**Authors:** Viktoria LINDBERG-LARSEN, Martin LINDBERG-LARSEN, Ole OVESEN, Stine T ZWISLER, Peter LINDHOLM, Stine HEBSGAARD, Robin CHRISTENSEN, Søren OVERGAARD

**Affiliations:** a Shared first authorship; 1 Department of Anesthesiology and Intensive Care Medicine, Odense University Hospital, Odense; 2 Section for Biostatistics and Evidence-Based Research, the Parker Institute, Bispebjerg and Frederiksberg Hospital, Copenhagen; 3 Department of Orthopedic Surgery and Traumatology, Odense University Hospital, Odense; 4 Orthopaedic Research Unit, Department of Clinical Research, University of Southern Denmark, Odense University Hospital, Odense; 5 Research Unit of Rheumatology, Department of Clinical Research, University of Southern Denmark, Odense University Hospital, Odense; 6 Department of Orthopedic Surgery and Traumatology, Copenhagen University Hospital, Bispebjerg Hospital, Copenhagen; 7 Department of Clinical Medicine, Faculty of Health and Medical Science, University of Copenhagen, Copenhagen, Denmark

## Abstract

**Background and purpose:**

Periacetabular osteotomy (PAO) for hip dysplasia is associated with intensive pain and high opioid consumption. High doses of dexamethasone may reduce this. We aimed to compare the effect of 1 or 2 doses of dexamethasone 24 mg, relative to placebo, on postoperative morphine consumption after PAO.

**Methods:**

A 3-group, randomized, double-blind, placebo-controlled trial was undertaken on patients ≥ 18 years, undergoing PAO (ClinicalTrials.gov: NCT03874936). Randomization Group A received 1 preoperative dose of dexamethasone 24 mg and placebo 24 hours later; Group B received 1 dose of intravenous dexamethasone 24 mg preoperatively and a repeated dose 24 hours postoperatively; and Group C received placebo at both time points. The primary endpoint was the difference in least squares mean cumulative postoperative morphine consumption between the combined dexamethasone groups and placebo within 48 hours from baseline. Key secondary outcomes included postoperative pain intensity, nausea and vomiting, antiemetic consumption and Timed Up and Go at 24 and 48 hours postoperatively, and cumulative morphine consumption from 48 hours to day 14 post-operation.

**Results:**

90 patients were randomized to dexamethasone groups (n = 60) and placebo (n = 30); 58 and 28, respectively, completed the trial. Mean age was 31 years and 71 (79%) were females. In the combined dexamethasone group the mean cumulated postoperative morphine consumption within 48 hours was 92 mg vs 95 mg in the placebo group, corresponding to a between-group difference of –3 mg (95% confidence interval –27 to 21; P = 0.8). There were no differences observed between groups for any of the secondary outcomes.

**Conclusion:**

High-dose dexamethasone did not reduce postoperative morphine use or improve any of the secondary outcomes after PAO.

Periacetabular osteotomy (PAO) is a procedure used to treat various hip-related conditions in young adults when non-surgical treatments are insufficient [1]. Most commonly, it is used for treating acetabular dysplasia characterized by insufficient bony coverage of the femoral head [2]. The procedure is performed to reduce pain, and improve function and quality of life. Moreover, it may also prevent development of secondary osteoarthritis and postpone the need for a total hip arthroplasty although there may still be a risk [3,4].

PAO involves release and reorientation of the acetabulum, which is associated with extensive surgical trauma of both muscles and bone [5], leading to a significant inflammatory stress response. Pain and nausea remain major postoperative challenges in this patient group [6], which is why high doses of opioids are administered, which further increases the risk of opioid-related side effects such as postoperative nausea and vomiting. High-dose glucocorticoids administered after total hip and knee arthroplasty have significantly reduced pain, postoperative nausea, and vomiting as well as length of hospital stay [7]. Specifically, 2 doses (preoperatively and 24 hours postoperatively) have shown beneficial effects [8]. Therefore, it is relevant to investigate whether high doses of glucocorticoids can also decrease opioid consumption, reduce postoperative nausea and vomiting, and alleviate pain in a young patient group undergoing an extensive surgical trauma such as in PAO.

The primary objective was to compare the effect of 1 or 2 high doses of dexamethasone (24 mg) with placebo on postoperative morphine consumption within 48 hours after PAO. We hypothesized that dexamethasone would decrease opioid consumption. The secondary aim was to evaluate the effect on pain scores, early postoperative function, prolonged opioid consumption, postoperative nausea and vomiting, and antiemetic consumption.

## Methods

This was a participant-, practitioner-, and outcome assessor-blinded, 3-arm, parallel-group, randomized, placebo-controlled trial conducted from November 2020 to December 2022.

An explicit Statistical Analysis Plan was performed prior to data analysis, ensuring systematic, unbiased analyses aligned with study objectives, particularly in the context of prespecified MockUps [9]. The final manuscript was prepared following the CONSORT statement [10]. The study protocol and the full Statistical Analysis Plan can be found in Supplementary data.

### Setting and eligibility criteria

Participants were recruited from the Department of Orthopedic Surgery and Traumatology, Odense University Hospital, Denmark. Inclusion criteria consisted of being 18 years or older, non-pregnant, and scheduled for PAO due to symptomatic hip dysplasia or retroverted acetabulum. Exclusion criteria included allergy or contraindications to trial medication, spinal anesthesia, second intervention carried out simultaneously (e.g., femoral osteotomy), daily opioid consumption prior to surgery (except tramadol and codeine), drug abuse, medical abuse defined as inappropriate/excessive use of drugs and/or prescribed medication, e.g., opioids, barbiturates etc. or weekly alcohol consumption beyond ≥ 7 units in females and ≥ 14 units in males, mental disability, anxiety disorder (active psychiatric disorder or consumption of tricyclic antidepressants), diabetes diagnoses prior to inclusion, and immune suppression therapy (e.g., systemic glucocorticoids).

### Surgical procedure

Surgery was performed by 3 experienced surgeons (SO, OO, MBØ) using the same modified Smith-Petersen approach and surgical technique [11]. No wound drains or local infiltration anesthesia were used. The procedure in brief begins with a skin incision at the anterior superior iliac spine and continues distally for approximately 7–10 cm. The fascia is incised and sartorius and psoas muscles are retracted. The periosteum is elevated along the medial aspect of the ilium, until it lies just below the linea terminalis and further medially to expose the pubic bone. The osteotomies are performed while the muscles are retracted. The acetabular fragment is fixed by 2 screws after the reorientation has been done.

### Standard anesthesia and analgesia

The patients received no pre-medication, and general anesthesia was used in all cases. Induction with propofol (10 mg/mL) 2–4 mg/kg followed by continuous administration of 5–15 mg/kg/hour, and remifentanil (50 µg/mL) 1 µg/kg followed by continuous administration of 0.5–1 µg/kg/minute. Basal fluid administration with crystalloids 2–3 mL/kg/h was administered, and severe bleeding was treated with balanced blood component therapy according to national guidelines [12]. Intravenous fentanyl loading was performed during surgery.

In the recovery room, a patient-controlled analgesia (PCA) pump with morphine bolus 0.04 mg/kg and lock-out of 8 minutes was initiated. Following local guidelines, a PCA bolus was recommended if VAS > 3. The PCA pump was continued for 48 hours. In the orthopedic ward, standard analgesic treatment consisted of tablet paracetamol 1 g 6 hourly, and tablet ibuprofen 400 mg 6 hourly (equal to 4 times daily). Moderate to severe postoperative nausea and vomiting was treated with antiemetics (ondansetron or droperidol) administered by ward nurses. In cases of insufficient effect of the PCA pump, rescue medication with alternative opioids (oxycodone) could be administered.

### Intervention

All patients scheduled for PAO were screened for eligibility in the outpatient clinic and provided with oral and written study information. Eligibility was reassessed at a pre-surgery visit, where final inclusion was confirmed. Randomization was performed within 48 hours before surgery, with trial drugs prepared by anesthetic personnel uninvolved in the study, following a concealed recipe in the randomization envelope. Identical, unmarked containers were then delivered to the operating theatre. Study medication (dexamethasone or placebo) was injected intravenously at induction of anesthesia prior to surgery and again 24 hours after surgery.

Participants were randomized to 1 of 3 groups:

- Dexamethasone (24 mg) at induction of anesthesia and placebo 24 hours after surgery.
- Dexamethasone (24 mg) at induction of anesthesia and dexamethasone (24 mg) 24 hours after surgery.
- Placebo (isotonic saline) at induction of anesthesia and placebo 24 hours after surgery.

### Randomization, treatment allocation, and blinding

Patients were randomized with a 1:1:1 allocation. An independent data manager developed the computer-generated list of random numbers using the randomization tool in Research Electronic Data Capture (REDCap; https://project-redcap.org/). The participants were randomized in permuted blocks of sizes 3 to 6 and the block sizes undisclosed to ensure concealment. Administrators of the randomization procedure were blinded to randomization at all times during the trial. The randomization code was stored in REDCap with no access by the author group or any other. All interventions were blinded to the participants, those administering the intervention, researchers, other care providers, and outcome assessors, as well as the statistician and investigators.

### Outcome measures and endpoints

Outcomes were measured within 48 hours after intervention for the primary and 9 of the 10 key secondary outcomes and at 14 days post-intervention for the remaining key secondary outcome. Adverse events within 8 weeks were registered.

The primary outcome was postoperative morphine consumption calculated in accumulated milligrams during 48 hours from baseline. Accordingly, the primary endpoint was the difference in least squares mean cumulative postoperative morphine consumption (mg) between the combined dexamethasone groups and placebo within 48 hours from baseline. All opioids administered postoperatively, including patient-controlled analgesia (PCA), i.v. morphine and other supplemental opioids, were converted to equivalent morphine doses.

The 10 key secondary outcomes were:

1. Postoperative pain intensity during rest at 24 hours. Pain intensity was assessed using the visual analogue scale (VAS) (0–100 mm).
2. Postoperative pain intensity during activity at 24 hours. Pain was evaluated under the Timed Up and Go (TUG) test using VAS.
3. Postoperative pain intensity during rest at 48 hours evaluated using VAS.
4. Postoperative pain intensity during activity at 48 hours evaluated under the TUG test using VAS.
5. Cumulated postoperative morphine consumption from 48 hours until day 14 post-operation.
6. Postoperative nausea and vomiting within 24 hours postoperatively. Nausea and vomiting were evaluated using a 4-point scale: none, mild, moderate, and severe.
7. Postoperative nausea and vomiting within 48 hours postoperatively.
8. Antiemetic consumption within 48 hours postoperatively.
9. TUG test at 24 hours postoperatively (the time that the participant takes to rise from a chair, walk 3 meters, turn around, walk back to the chair, and sit down).
10. TUG test at 48 hours postoperatively.

According to the Statistical Analysis Plan, the secondary objectives will only be outlined to include comparison of all 3 intervention groups if statistically significant differences between placebo and the combined groups of dexamethasone are found.

### Sample size and power calculation

A pilot study including 2 patients receiving 24 mg dexamethasone preoperatively and 8 patients treated with care as usual was conducted to gather empirical data, revealing an average of 47 mg of morphine consumption within 48 hours post-PAO, with a standard deviation (SD) of 33 mg. In the pilot study the mean (SD) in the placebo and dexamethasone group corresponded to 57 (32) and 20 (14) mg morphine, respectively. This indicated a potential effect of adding dexamethasone corresponding to an estimated treatment difference of promising 37 mg morphine per se (i.e., potential net benefit). Hence, for a 2-sample pooled t-test of a normal mean difference with a 2-sided significance level of 0.05, assuming a common standard deviation of 40 mg morphine, a total sample size of 87 patients assuming an allocation ratio of 2 to 1 (corresponding to group dexamethasone/placebo and dexamethasone/dexamethasone vs group placebo/placebo) was required to obtain a power of 90% to detect an estimated treatment difference of 30 mg morphine. Based on this, it was decided to aim for enrolment of 90 participants in total in the intention-to-treat population (randomization: 30:30:30; i.e., n_A_ + n_B_ = 60 vs n_C_ = 30).

### Statistics

The primary analyses were performed on the intention-to-treat (ITT) population. Thus, all data from individuals randomized, regardless of dropout and participation, was included in the main analysis belonging to the initially allocated. The prespecified Statistical Analysis Plan (SAP) was finalized prior to the commencement of any data analysis (see Supplementary data). Statistical testing for baseline imbalance is considered inappropriate in randomized trials [13]. Baseline characteristics are summarized and interpreted clinically, as any observed imbalance is assumed to be due to chance. Consequently, hypothesis testing of baseline characteristics was deemed unnecessary as they may mislead.

Continuous endpoints were analyzed using an analysis of covariance (ANCOVA) model with randomized treatment group as a factor, and the baseline endpoint value as a covariate. Categorical endpoints were analyzed using logistic regression, with treatment group as a factor, and the baseline endpoint value as a covariate. For the main analyses, missing data was handled using multiple imputation procedures in SAS software (SAS Institute, Cary, NC, USA), employing the Markov Chain Monte Carlo (MCMC) method. The model included all patient demographics, clinical measurements, and other relevant variables; in the context of multiple imputation, a “VAR statement” was used to specify the variables to be included in the imputation model—that is, the variables defining the multivariate distribution used in the imputation process. Missing data was imputed 5 times within each randomized treatment group, and the results were combined using Rubin’s rules [13]. For the continuous outcomes, the results are reported for each group as least squares means with standard errors, while the differences between groups are reported with 95% confidence intervals (CI) [13].

The primary null hypothesis was based on the comparison between participants randomized to any dose of dexamethasone (group A&B) vs placebo (group C); H0: μA+B = μC. We specified that a CI excluding differences greater than 10 mg of morphine between groups should be interpreted as indicating the absence of a clinically meaningful difference.

**Figure:**
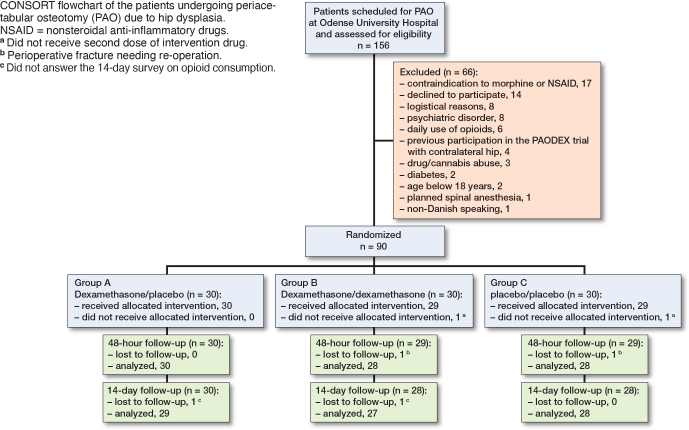


All reported P values and 95% CIs are 2-sided and not adjusted for multiple comparisons by default. The statistical significance was set at the conventional level of α < 0.05 (P < 0.05). All analyses were performed using commercially available statistical software (i.e., SAS Studio).

### Ethics, registration, data sharing, funding, and disclosures

Approval was obtained from the Regional Committees on Health Research Ethics for Southern Denmark (Project-ID S-20190017) on April 3, 2019 and the Danish Data Protection Agency (Journal No 19/10040). All participants provided oral and written informed consent in accordance with the Declaration of Helsinki. The trial adhered to the Declaration of Helsinki, the International Conference on Harmonization guidelines, and Good Clinical Practices. The trial protocol followed the SPIRIT statement [14]. Before patient enrollment, the trial was registered at ClinicalTrials.gov (NCT03874936) in March 2019. Anonymized data from this study may be available from the corresponding author upon reasonable request. The study received financial funding from the Odense University Hospital Free Research Fund. The Section for Biostatistics and Evidence-based Research, the Parker Institute, Bispebjerg and Frederiksberg Hospital is supported by a core grant (OFIL-24-074) from the Oak Foundation. The authors report no conflicts of interest. Complete disclosure of interest forms according to ICMJE are available on the article page, doi: 10.2340/17453674.2025.43903

## Results

### Disposition and baseline characteristics of participants

Between November 2020 and December 2022, 156 individuals scheduled for PAO surgery were screened for eligibility and 90 participants signed informed consent, were enrolled and randomized, and thus constituted the ITT population (Figure). The last patient visit was in February 2023. A total of 60 participants were randomized to 1 of the 2 dexamethasone groups (1 dose or 2 doses of dexamethasone 24 mg) and 30 participants were randomized to the placebo group (2 doses of placebo). Mean age was 31 years and 71 (79%) were females. There were a lower proportion of ASA group II patients in the dexamethasone groups of 30% compared with 12% in the placebo group. Otherwise baseline patient and intraoperative characteristics were similar between the groups (Table 1). None of the included patients used tramadol or codeine preoperatively.

**Table 1:** Demographic and clinical characteristics of the intention-to-treat population of patients undergoing PAO due to hip dysplasia at baseline. Values are means (SD), unless otherwise stated

Characteristic	Dexamethasone groups (A+B) (n = 60)	Placebo group (C) (n = 30)	Combined (n = 90)
Female sex, n (%)	49 (82)	22 (73)	71 (79)
Age, year	30 (9)	33 (11)	31 (9)
Height, cm	171 (9)	172 (8)	172 (9)
Weight, kg	74 (14)	74 (9)	74 (12)
Body mass index	25 (3)	25 (3)	25 (3)
ASA physical-status classification, n (%)			
Class I	53 (88)	21 (70)	74 (82)
Class II	7 (12)	5 (30)	16 (18)
Current smoker, n (%)	9 (15)	4 (13)	13 (15)
Alcohol units/week, n (%)			
0	24 (41)	13 (43)	37 (42)
1–7	32 (54)	12 (40)	44 (49)
8–14	3 (5.0)	5 (17)	8 (8.9)
> 14	0	0	0
Side of surgery, dexter, n (%)	34 (57)	16 (55)	50 (56)
Duration of surgery, median (IQR), min	78 (69–93)	81 (74–91)	80 (39–91)
Fluid administration, median (IQR), L	1.3 (1.1–1.5)	1.3 (0.8–1.5)	1.3 (1.0–1.5)
Bleeding, median (IQR), mL	540 (425–770)	525 (350–700)	540 (400–750)
Propofoll ^a^, median (IQR), mg	919 (792–1,038)	849 (751–941)	896 (762–1,019)
Remifentanil ^a^, median (IQR), g	3.3 (2.7–4.1)	3.3 (2.3–4.1)	3.3 (2.5–4.1)
Fentanyl ^a^, median (IQR), µg	600 (500–600)	525 (500–650)	550 (500–600)
VAS, recovery room, median (IQR)	60 (35–80)	60 (30–80)	60 (30–80)

a Cumulated dose.

VAS = visual analogue scale (points 0–100), pain evaluation

Percentages may not sum to 100 because of rounding.

### Primary and key secondary outcomes

The mean cumulated postoperative morphine consumption within 48 hours postoperatively was 92 mg (standard error [SE] 7) in the combined dexamethasone group vs 95 mg (SE 10) in the placebo group, corresponding to a between-group difference of –3 mg (CI –27 to 21; P = 0.8). (Table 2). Among the 10 key secondary outcomes there were no statistically significant differences observed between the combined dexamethasone group and the placebo group at any time point (Table 2).

**Table 2:** Primary and 10 key secondary outcomes of the intention-to-treat population of patients undergoing PAO due to hip dysplasia

Characteristic	Dexamethasone groups (A+B) (n = 60)	Placebo group (C) (n = 30)	Difference (CI)	P value
Primary outcome				
Cumulated postoperative morphine consumption, up to 48 hours, mg	92 (7)	95 (10)	–3 (–27 to 21)	0.8
Key secondary outcomes (postoperative)^a^				
Pain intensity after 24 hours				
VAS at rest	35 (3)	34 (4)	0.8 (–8 to 10) 0.9	
VAS during activity	38 (4)	37 (5)	2 (–11 to14)	0.8
Pain intensity after 48 hours				
VAS at rest	39 (3)	39 (4)	–0.1 (–9 to 9)	1
VAS during activity	42 (3)	46 (4)	–4 (–14 to 6)	0.5
Cumulated morphine from				
48 hours to 14 days, mg	157 (23)	168 (32)	–11 (–88 to 66)	0.8
Nausea and vomiting (points 0–3)				
at 24 hours	0.7 (0.1)	0.9 (0.2)	–0.3 (–0.7 to 0.1)	0.2
at 48 hours	0.6 (0.1)	0.8 (0.2)	–0.2 (–0.6 to 0.2)	0.3
Cumulated antiemetic consumption				
at 48 hours, mg	5 (0.8)	8 (1.2)	–2 (–5 to 0.6)	0.1
Timed up and go test, s				
at 24 hours	123 (11)	132 (15)	–9 (–45 to 27)	0.6
at 48 hours	44 (4)	41 (5)	3 (–9 to15)	0.6

VAS = visual analogue scale (points 0–100)

a Reported for each group as least squares means with standard errors while the difference between them is reported with 95% confidence intervals (CI). Continuous endpoints were analyzed using an analysis of covariance (ANCOVA) model with randomized treatment and baseline outcome value as covariate. Missing data was imputed from all retrieved patients and the results were combined using Rubin’s rules.

### Harms and adverse events

2 participants (1 in the combined dexamethasone group and 1 in the placebo group) needed reoperation in the early postoperative period. One patient had an unintended intraoperative fracture and the second patient had a displaced osteotomy due to a fall on the ward. Both patients underwent secondary surgery with osteosynthesis during the primary admission. No other serious adverse events were registered within 8 weeks postoperatively (Table 3).

**Table 3:** Reporting of harms in patients undergoing PAO due to hip dysplasia. Values are numbers and (percentages)

Characteristic	Dexamethasone/placebo (A) (n = 30)	Dexamethasone/dexamethasone (B) (n = 30)	Placebo (C) (n = 30)
Serious adverse events (SAE), up to 8 weeks after operation	0	1 (3.3)^a^	1 (3.3)^b^
Deep infection	0	0	0
Revision due to acetabular/femoral fracture	0	1 (3.3)^a^	1 (3.3)^b^
Deep venous thrombosis/pulmonary embolism	0	0	0
Nerve injury	0	0	0
Death	0	0	0

a Displaced osteotomy due to a fall on the ward requiring reoperation .

b Unintended intraoperative fracture requiring reoperation.

## Discussion

Compared with placebo, this trial found that 1 or 2 doses of 24 mg dexamethasone did not reduce opioid consumption within 48 hours after PAO. Additionally, there was no effect on patient-reported pain scores, prolonged opioid consumption (2–14 days postoperatively), postoperative nausea and vomiting, antiemetic consumption, or early function.

These findings align with the only other previous randomized controlled trial investigating the effect of 1-dose glucocorticoids after PAO [6]. Although Steinthorsdottir et al. [6] compared a larger dose (48 mg) of dexamethasone with 8 mg and used patient-reported pain scores in the post-anesthesia care unit as primary endpoint, the signal of no effect of high-dose dexamethasone on postoperative pain in this patient group was similar. However, Steinthorsdottir et al. indicated a potential effect of 48 mg of dexamethasone on cumulative opioid use over 4 days, but this could not be confirmed in our study at 2 or 14 days postoperatively. Differences in study designs may explain this discrepancy, as our study focused on opioid consumption using a PCA pump as the primary endpoint, while Steinthorsdottir et al. had opioid consumption as a secondary outcome not using a PCA pump. Furthermore, Steinthorsdottir et al. found lower rescue opioid consumption only on postoperative day 1 in the high-dose group. Thus, our findings, combined with those of Steinthorsdottir et al., confirm that high-dose dexamethasone (1 or 2 doses) has limited or no effect on postoperative pain and opioid consumption in patients undergoing PAO.

Younger patients might be able to generate a more intense inflammatory response to surgery compared with elderly patients and should in theory benefit from anti-inflammatory treatment. Therefore, the lack of effect of high-dose dexamethasone on opioid consumption after PAO surgery was unexpected, given its documented benefits on postoperative opioid consumption and pain in other orthopedic procedures such as TKA and THA [7,8]. The effectiveness of high-dose dexamethasone on pain in TKA and THA has been demonstrated in even relatively small RCT-cohorts (n = 2 x 24) [15,16]. Theoretically, TKA, and especially THA, might surgically be considered less extensive than PAO. Additionally, TKA and THA are mainly performed on older patients with a mean age of around 68 years. The inflammatory response in the elderly was in previous studies found to be increasingly variable and less pronounced compared with younger patients [17]. Hence, it was surprising that 1 or 2 high doses of dexamethasone showed no effect on younger PAO patients with a mean age of 31 years. This might be explained by the fact that the intense inflammatory response and associated pain in young, otherwise healthy patients cannot be adequately reduced by glucocorticoids. Further exploration of the systemic inflammatory response to PAO and its biochemical effects is necessary to understand this discrepancy [18].

Because high-dose glucocorticoids do not appear to provide the same benefits in terms of postoperative pain relief and opioid-sparing effects following PAO as seen after hip [15] and knee arthroplasty [8], alternative strategies must be considered. A shift in anesthetic technique to spinal anesthesia combined with deep sedation and extended multimodal analgesic regimens—potentially including adjuncts such as methadone or S-ketamine—may help reduce postoperative pain and enhance mobilization. However, these approaches require evaluation in future clinical trials.

### Strengths and limitations

The strengths of the present study include the randomized and blinded design, and the high degree of standardization regarding the perioperative setting; all patients received standardized general anesthesia. Perioperative fluid management was registered in detail, and the perioperative analgesic regimen was standardized as well. Surgery was performed by only a few experienced surgeons in a single-center setup. Furthermore, the patient characteristics of the patients included are similar to PAO patients in general [2], increasing generalizability of the study. Limitations include the possibility that some patients minimized the use of the PCA pump due to side effects such as dizziness and the sensation of a “rush” from intravenous morphine. However, average morphine consumption within 48 hours postoperatively was substantially higher in the trial (> 93 mg) than in the pre-trial pilot (47 mg), suggesting participants used PCA to meet actual analgesic needs rather than relying on nurse-administered doses. Although morphine consumption was higher than expected based on data from the pilot study, another limitation is the potential for a type 2 error but, given the lack of any signal of a positive effect of dexamethasone (1 or 2 doses) on opioid consumption or secondary outcomes, it is unlikely that a larger cohort would reveal a clinically relevant effect. Our findings raise more questions than answers, emphasizing the need for a better understanding of systemic inflammatory and pain responses to optimize perioperative analgesia in young patients undergoing extensive surgical trauma.

### Conclusion

We could not demonstrate any effect of 1 or 2 doses of high-dose dexamethasone compared with placebo on reduction of postoperative opioid consumption within 48 hours or other secondary outcomes in young patients undergoing PAO surgery.

*In perspective,* our results emphasize that the positive effects of high-dose glucocorticoids seen in the most common and standardized orthopedic procedures, like THA and TKA, do not apply to all orthopedic surgeries. The limited sample size may have contributed to a type 2 error, as the 95% confidence interval was not narrow enough to exclude a difference exceeding the prespecified 10 mg morphine threshold for clinical relevance. Therefore, high-dose glucocorticoids and other alternative analgesic strategies should be evaluated more thoroughly before implementation.

### Supplementary data

The Statistical Analysis Plan is available as supplementary data on the article page, doi: 10.2340/17453674.2025.43903

## Supplementary Material





## Notes

VLL was primary investigator. SO, OO, SZ, and PL initiated the study and OO included most patients in the study and performed most of the PAO procedures. SH wrote the first protocol draft, which was finished by VLL. The statistical analysis plan was written by VLL and MLL and supervised by RC. RC performed the statistical analyses. MLL and VLL wrote the first draft of the manuscript, which was later critically reviewed by all co-authors.

The authors would like to thank all patients who participated, as well as the orthopedic surgeons, anesthetists, and the local trial coordinator, Annie Gam-Pedersen. They also extend their gratitude to the nurses, physiotherapists, and the Department of Orthopedic Surgery and Traumatology at Odense University Hospital for their support with outcome assessments. Additionally, they acknowledge the Odense Patient Data Explorative Network for data management assistance and access to REDCap software, and Henriette Kunoy Bendixen from the GCP Unit for her support.

Handling co-editors: Paul Gerdhem and Philippe Wagner

*Acta* thanks Katarina Greve and Linda Helenius for help with peer review of this manuscript.

## Appendix

**Table:** Exploratory 3-group analyses of the primary and 10 key secondary outcomes of the intention-to-treat population of patients undergoing PAO due to hip dysplasia

Characteristic	Dexamethasone/placebo (A) (n = 30)	Dexamethasone/dexamethasone (B) (n = 30)	Placebo (C) (n = 30)
Primary outcome			
Cumulated postoperative morphine consumption, up to 48 hours, mg	90.2 (9.9)	93.8 (9.9)	94.8 (9.9)
Key secondary outcomes (postoperative)^a^			
Pain intensity after 24 hours			
VAS at rest	32.0 (3.7)	38.4 (3.7)	34.4 (3.7)
VAS during activity	35.0 (5.1)	41.5 (5.1)	36.8 (5.1)
Pain intensity after 48 hours			
VAS at rest	36.4 (3.7)	41.6 (3.7)	39.2 (3.7)
VAS during activity	38.8 (4.2)	45.2 (4.2)	45.8 (4.2)
Cumulated morphine from			
48 hours to 14 days, mg	180.6 (32.0)	134.0 (32.0)	168.3 (32.0)
Nausea and vomiting (points 0–3)			
at 24 hours	0.8 (0.2)	0.6 (0.2)	0.9 (0.2)
at 48 hours	0.7 (0.2)	0.4 (0.2)	0.8 (0.2)
Cumulated antiemetic consumption			
at 48 hours, mg	6.1 (1.2)	4.8 (1.2)	7.8 (1.2)
Timed up and go test			
at 24 hours, s	117.1 (14.8)	129.5 (14.8)	132.3 (14.8)
at 48 hours, seconds	39.1 (5.0)	48.9 (5.0)	41.1 (5.0)

VAS = visual analogue scale (points 0–100).

a Reported for each group as least squares means with standard errors.
