# Supplementary material for: Effect of high-dose dexamethasone on morphine use after periacetabular osteotomy for hip dysplasia: a randomized double-blind placebo-controlled single center trial
Source: Acta Orthop. 2025 Jun 1;96:413–20. doi: 10.2340/17453674.2025.43903 (PMC12128623; doi:10.2340/17453674.2025.43903)
Supplement: Supplementary file 1 [file ActaO-96-43903-s1.pdf]

PROTOCOL

The effect of perioperative dexamethasone administration on postoperative pain in patients undergoing periacetabular osteotomy: *A randomised double-blind, placebo-controlled trial*

The PAODEX trial

Version: 4.0_20210507
Date: May 7th 2021

CONFIDENTIAL

This document is confidential and the property of Odense University Hospital and the association between Dept. of Orthopaedic Surgery and dept. Anaesthesiology and Intensive Care. No part of it may be transmitted, reproduced, published, or used without prior written authorisation from the institution.

Statement of Compliance

This document is a protocol for a research project. This study will be conducted in compliance with all stipulation of this protocol, the conditions of the ethics committee approval, the NHMRC National Statement on ethical Conduct in Human Research (2007) and the Note for Guidance on Good Clinical Practice (CPMP/ICH-135/95).

The undersigned Sponsor and Primary investigator confirm hereby to follow the protocol and work after the guidelines for Good Clinical Practice.

It is confirmed that in accordance with Danish law respectively the GCP unit and other relevant authorities have access to source data/documents including patient files connection with monitoring, auditing as well as upon inspection.

The protocol follows international guidelines from Standard Protocol Items: Recommendations for International Trials (SPIRIT)[1]. The reporting of the trial will follow the CONSOlidated standards of Reporting Trials (CONSORT)[2].

Sponsor and primary investigator
Viktoria Lindberg-Larsen, MD, PhD, Department of Anaesthesiology and Intensive Care, Odense University Hospital.

Date: Signature: .

Investigator:
Stine T. Zwisler, MD, PhD, Consultant, Department of Anaesthesiology and Intensive Care, Odense University Hospital.

Date: Signature: .

Senior biostatistician responsible:
Robin Christensen, BSc, MSc, PhD, Professor of Biostatistics and Clinical Epidemiology; Research Unit of Rheumatology, Department of Clinical Research, University of Southern Denmark, Odense University Hospital, Denmark.

Date: Signature: .

CONTENTS

CONTENTS **3**

ADMINISTRATIVE INFORMATION **5**

1. TITLE 5
2. TRIAL REGISTRATION AND APPROVALS 5
3. PROTOCOL VERSION 5
4. FUNDING 5
5. ROLES AND RESPONSIBILITIES 6
   5a Contributor-ship *6*
   5b Sponsor Contact Information *7*
   5c Sponsor and Funder *8*
   5d Committees *8*
   5E Timeplan for Trial: *8*

INTRODUCTION **9**

6. BACKGROUND AND RATIONALE 9
   6a Choice of Comparators *9*
   6B Pathophysiology And Glucocorticoids: *9*
   6c Population *10*
   6d Evidence-Based Research: *10*
   6E Motivation: *10*
   6F Dose and Drug Selection, including Potential Risk/Benefits *11*
   6g Explanation for Choice of Comparators *11*
7. OBJECTIVES AND HYPOTHESIS 12
   Research hypothesis: *12*
   0-hypothesis *12*
8. STUDY DESIGN 12
   Study Timeline (flow diagram) *13*

METHODS: PARTICIPANTS, INTERVENTIONS, OUTCOMES **15**

9. STUDY SETTING 15
10. ELIGIBILITY CRITERIA 15
11. INTERVENTIONS 15
   Interventional drug: *15*
   11A Interventions *16*
   11b Modifications *16*
   11c Adherence *17*
   11d Concomitant Care *17*
12. OUTCOMES 19
13. PARTICIPANT TIMELINE 20
14. SAMPLE SIZE AND POWER CONSIDERATIONS 20
15. RECRUITMENT 21

METHODS: ASSIGNMENT OF INTERVENTIONS **21**

16. ALLOCATION 21
   16a Sequence Generation *21*
   16b Concealment Mechanism *21*
   16c Implementation *21*
17. BLINDING (MASKING) 22
   17a Blinding (masking) *22*
   17b Emergency Unblinding *23*

METHODS: DATA COLLECTION, MANAGEMENT, ANALYSIS **23**

18 DATA COLLECTION METHODS 23
   18a Data Collection Methods *23*
   18b Retention *26*

19 DATA MANAGEMENT 26
20 STATISTICAL METHODS 26
20a Primary analyses 26
20b Additional analyses 27
20c missing data and reporting of protocol deviations 28

METHODS: MONITORING 29

21 DATA MONITORING 29
21A Formal Committee 29
21b Interim Analysis 29
22 HARMS - ADVERSE EVENTS 29
23 AUDITING AND QUALITY ASSURANCE 31

ETHICS AND DISSEMINATION 31

24 RESEARCH ETHICS APPROVAL 31
24A Ethical considerations 31
25. PROTOCOL AMENDMENTS 31
26. CONSENT 31
27. CONFIDENTIALITY 32
28. DECLARATION OF INTERESTS 33
29. ACCESS TO DATA 33
30. ANCILLARY AND POST-TRAIL CARE 33
31. DISSEMINATION POLICY 34
31a Trial Results and Dissemination Policy 34
31b Authorship 34
31c Reproducible Research 34

APPENDICES 35

32. INFORMED CONSENT MATERIALS 35
32a Other documents 35
33. BIOLOGICAL SPECIMENS 35
34. REFERENCES: 36

ADMINISTRATIVE INFORMATION

1. TITLE

The effect of perioperative dexamethasone administration on postoperative pain in patients undergoing periacetabular osteotomy: A randomised double-blind, placebo-controlled trial

Short title:
PAODEX (PeriAcetabular Osteotomy and DEXamethasone)

2. TRIAL REGISTRATION AND APPROVALS

ClinicalTrials.gov: NCT03874936
EudraCT: 2019-000402-30
Danish Medical Agency: 2019030309
Research Ethics Committee of Region of Southern Denmark: S-20190017
The Danish Data Protection Agency: 19/10040

3. PROTOCOL VERSION

Version: 4.0_20210507

4. FUNDING

South Danish Tissue Centre is funding the buyout for protocol development, implementation and costs for application fees to the Danish Medical Agency and the Research Ethics Committee of Region of Southern Denmark up to DKK 232.500. The grant is placed at an account no: 102 -203-090-001, administered by the research administration at Odense University Hospital.

Proposed sources of financial support:
We intend to apply for:

1. Funds for free research by Odense University Hospital:
   Wages costs of study nurse
2. Funds by the department of Orthopaedic Surgery, Odense University Hospital:
   Costs of study medicine
3. Funds by the department of Anaesthesiology and Intensive Care, Odense University Hospital:
   Cases for PCA-pumps and morphine
4. Funds by DASAIM and others:
   Promotion, services and other costs
5. Other private and public funds.

Budget, see Appendix 1.

5. Roles and Responsibilities

5a Contributor-ship

Trial Sponsor until May 2021:
Name: Søren Overgaard (SO)
Title: Head of research, Professor, DMSc.
Affiliation: Department of Orthopaedic Surgery and Traumatology, Odense University Hospital, Department of Clinical Research, University of Southern Denmark.

Primary and corresponding investigator until May 2019:
Name: Stine Hebsgaard (SH)
Title: MD
Affiliation: Department of Anaesthesiology and Intensive Care, Odense University Hospital

Primary Investigator after May 1st 2019 and sponsorinvestigator from May 2021
Name: Viktoria Lindberg-Larsen (VLL)
Title: MD, PhD.
Affiliation: Department of Anaesthesiology and Intensive Care, Odense University Hospital

Statistical Advisor:
Name: Robin Christensen (RC)
Title: BSc, MSc, PhD, Professor of Biostatistics and Clinical Epidemiology
Affiliation: Research Unit of Rheumatology, Department of Clinical Research, University of Southern Denmark, Odense University Hospital, Denmark.

Investigators:
Name: Stine T. Zwisler (STZ)
Title: MD, PhD, Consultant
Affiliation: Department of Anaesthesiology and Intensive Care, Odense University Hospital

Name: Peter Lindholm (PL)
Title: MD, Consultant
Affiliation: Department of Anaesthesiology and Intensive Care, Odense University Hospital

Name: Ole Ovesen (OO)
Title: MD, Consultant
Affiliation: Department of Orthopaedic Surgery and Traumatology, Odense University Hospital.

Name: Morten Bøgehøj (MB)
Title: MD, PhD, Consultant
Affiliation: Department of Orthopaedic Surgery and Traumatology, Odense University Hospital.

Trial nurse:
Name: Annie Gam Pedersen (AGP)
Affiliation: Department of Orthopaedic Surgery and Traumatology, Odense University Hospital.

All investigators contributed to conceiving of the study. All investigators participated in elaboration of method section. SH elaborated the study protocol. All investigators will help with implementation of the study. AGP will accomplish the randomization. RC will provide statistical expertise in clinical trial design and will be involved in the primary statistical analyses. All authors contributed to refinement of the study protocol and will approve the final manuscript.

Collaborators:
Monitor, on behalf of sponsor:

GCP-unit, Odense University Hospital
Co. Henriette Kunoy Bendixen, cand.pharm.
J. B. Winsløws Vej 19, 2. floor, 5000 Odense C
hkbendixen@health.sdu.dk, tel. +45 6550 3705

Clinical Biochemistry and Pharmacology

Co. Mette Andreasen, project biomedical laboratory scientist
J. B. Winsløws Vej 4, 1. floor, room A, 5000 Odense C, Denmark.
Mette.andreasen@rsyd.dk, tel. +45 2170 5430

Other delegated from the department, responsible for cytokine analyses.

OPEN – Odense Patient data Explorative Network

OUH – Odense University Hospital Region of Southern Denmark
J. B. Winsløws Vej 9, 3 Floor
open@rsyd.dk

5B SPONSOR CONTACT INFORMATION

**Study Sponsor (until May 2021)**	**Søren Overgaard**<br>Department of Orthopaedic Surgery,<br>Odense University Hospital
**CVR. No:**	30 04 91 79
**Sponsor, E-mail**	Soeren.Overgaard@rsyd.dk
**Sponsorinvestigator from May 2021**	**Viktoria Lindberg-Larsen**<br>Department of Anaesthesiology and Intensive Care, Odense University Hospital<br>viktoria.lindberg-larsen@rsyd.dk
**Contact Name**	**Viktoria Lindberg-Larsen**
**Address**	J.B. Winsløws Vej 4, entrance 7-8, DK-Odense C, Denmark
**Telephone**	Mobile: +45 28791991
**E-mail, corresponding investigator**	viktoria.lindberg-larsen@rsyd.dk

5C SPONSOR AND FUNDER

The funding source, South Danish Tissue Centre had no role in the design of this study and will not have any role during execution of the study, analyses and interpretation of the data, as well as decision to publication.

5D COMMITTEES

- Primary Investigator:
  - Responsible for conduction of PAODEX.
- Corresponding and Primary investigator:
  - Responsible for preparation of protocol and revisions. Organizing of project group meetings.
- Project Group:
  - Consists of: VLL, SH, SO, STZ, OO, PL, MB, RC.
  - Agreement of final protocol including outcomes. Study planning. Recruitment of patients. Reviewing progress of study any if necessarily agreeing changes to the protocol and/or investigators brochure to facilitate the smooth running of the study.
  - Budget administration, randomisation, and data verification.

5E TIMEPLAN FOR TRIAL:

- February 2018 – March 2020: Preparation of trial protocol and trial documents for PAODEX, and registration of trial, applications for funds.
- April 2020 – December 2021: Enrolment and execution of PAODEX and sample analyses.
- January 2022 – December 2022: Data processing and reporting.

INTRODUCTION

6. BACKGROUND AND RATIONALE

Project summary – see appendix 2
Abbreviation list – see appendix 12

6A CHOICE OF COMPARATORS

Effective early postoperative multimodal pain management promoting early mobilisation, fluid and food intake, and recovery of normal activities is essential for wellbeing and rehabilitation of surgical patients[3].

Recently, an unpublished local observational pilot study on 10 patients indicated that periacetabular osteotomy (PAO) for hip dysplasia is associated with considerable pain treatment and nausea. A supplemental unpublished local pilot study on patient reported endpoint measures indicated that postoperative nausea and vomiting (PONV) was highly rated by the included patients. Pain rated just below PONV. Therefore, there is a great need for optimisation on pain and PONV treatment.

To manage pain different regimes may be considered; repeated local infiltration in combination with oral opioids showed no superiority to solitary opioids within 48 hrs. To our knowledge, other regimens using continuous lumbar epidural analgesia or paravertebral psoas blockade are to our knowledge not yet investigated in this population in prospective randomized trials. Opioids are associated with complicating side effects such as nausea, vomiting, drowsiness and respiratory depression[4]. Poor postoperative pain management and opioid side effect related malaise lead to prolonged time to resumption of fluids and food intake. Moreover, risk of prolonged immobilisation is known to increase the risk of deep vein thrombosis, though the crude incidence for development of venous thromboembolism has been reported to be low (0.94%)[5].

There is a need for effective postoperative pain management with a minimum of side effects. Several strategies to manage pain and PONV has been suggested of which glucocorticoids are gaining ground. To our knowledge there is yet no evidence supporting the use of short-term high dose intravenous glucocorticoid for postoperative analgesia after PAO.

6B PATHOPHYSIOLOGY AND GLUCOCORTICOIDS:

There is strong evidence supporting the use of glucocorticoids in multimodal analgesia protocols to optimize postoperative recovery and reduce opioid consumption and related side effects[6], in particular surgery with substantially trauma, pain and oedema. Gradually, the use of glucocorticoids for postoperative pain relief gain acceptance, though concerns regarding side effects as adrenal suppression, osteonecrosis, impaired wound healing and efficacy influence the application. Adrenal suppression is well described in literature during short-term use of glucocorticoids, however, this suppression is a clinically benign and reversible condition[7,8]. Well known anti-inflammatory properties of glucocorticoids have repeatedly shown significant reduction in the postsurgical inflammatory response after orthopaedic surgery[9–12].

Short-term perioperative administration of glucocorticoids is considered safe and low risk, including wound infection, hyperglycaemia or severe adverse events [13–17]. Though it is well established that glucocorticoids can elevate the blood glucose levels in patients (both with and without diabetes). One or two consecutive doses peri- and postoperatively, did lead to significant, but clinically irrelevant elevations of blood glucose (approximately 1-2 mmol/l[16]), and no adverse effects was linked to this risk[16,17].

6C POPULATION

Hip dysplasia typically manifests itself in adolescence or young adulthood, after decades of subclinical development [18,19]. The prevalence of radiographically hip dysplasia has been found to be 5% in both females and males of which not all are symptomatic[20]. A resent epidemiologic study reported that 83% of patients undergoing PAO were of female gender with mean age of 25.5 years[21]. Generally, this populations has a low grade of severe comorbidities[21].

Symptomatic hip dysplasia is shown to reduce physical activity as walking, and increase self-reported pain scores [21,22]. Hip dysplasia significantly increase the risk of developing secondary hip osteoarthrosis, why this population is offered PAO[18,19], as PAO outcome studies have demonstrated major clinically important improvements in pain, function, quality of life and activity level[23].

6D EVIDENCE-BASED RESEARCH:

In total knee and hip arthroplasty perioperative administration of a single dose glucocorticoid has been reported to reduce pain and PONV[15]. A recent meta-analysis on total joint arthroplasty reports significant reduction in PONV and pain at 12, 24 and 48hrs postoperatively after a single dose of glucocorticoid, most significantly at 12 and 24 hrs[13]. Other reported benefits include reduced pain during activity and improved mobility after surgery[10]. Furthermore, a single study on total joint arthroplasty shows extended effect on pain and opioid consumption after a repeated dose of dexamethasone (DXMT) 24hrs postoperatively compared to a single dose [10]. Recently, the need for further research into repeated administration of glucocorticoids to improve postoperative pain after knee arthroplasty was suggested[24]. The knee and hip arthroplasty populations are markedly older with more comorbidities than hip dysplasia patients[25,26]. Several reports conclude that more and larger RCTs concerning efficacy and safety are warranted before making final recommendations on perioperative use of glucocorticoids [6,13,15,24].

6E MOTIVATION:

In spite of careful attention to perioperative pain management after PAO pain and nausea remain a major challenge to the patients. The sizable need for opioids increase the risk of opioid-related side effects in a low-comorbidity population already in increased risk of PONV[27]. Therefore, it is relevant to investigate regimens to reduce opioid consumption and PONV.

To our knowledge, no experience with glucocorticoids as analgesic in PAO patients exists. As the PAO-procedure leads to major surgical trauma and pain, we consider it likely

that these patients will benefit from glucocorticoid administration, based on the assumption that perioperative glucocorticoid treatment in other orthopaedic procedures with major trauma has shown significant reduction in pain and PONV.

A regime including a repeated postoperative glucocorticoid administration is expected to result in a clinically relevant reduction in opioid consumption and opioid-related side effects. Generally, analgesic regimes including administration of repeated glucocorticoid doses are not sufficiently investigated.

6F Dose and Drug Selection, including Potential Risk/Benefits

A recent systematic review evaluates glucocorticoids for pain management in total joint arthroplasty. The included RCTs use different types of glucocorticoids; prednisolone, methylprednisolone, hydrocortisone and DXMT[15]. After converting the trial doses for comparison, we decided to use a high-dose DXMT of 24mg, as we wanted to evaluate the efficacy on pain, requiring larger doses compared to PONV management.

Furthermore, we selected DXMT based on the known postoperative pattern of pain, with highest intensity on postoperative day (POD)4[28]. DXMT is categorised as a long-acting glucocorticoid with a biologic half-life of 36-54hrs, with superior anti-inflammatory potency compared to other glucocorticoids[29]. A repeated dose on POD1, is intended to cover in the most pain-intense period.

Based on present evidence this trial should not involve risk of adverse effects exceeding those considered normal for this surgical procedure. Generally, DXMT is well tolerated and is standard use in high-risk PONV patients, though in lower doses. For *long term* use DXMT has multiple adverse effects[30].

In this trial the treatment period is short-term (0-24 hrs postoperatively), and the risk of serious adverse events are considered low[30]. We believe that participation in this trial is associated with very low risks short as long term.

We hope that patients receiving the intervention will benefit from the analgesic and anti-inflammatory effects of DXMT, including better mobilization and rehabilitation.

6G Explanation for Choice of Comparators

Previously, glucocorticoids have not been investigated in a population comparable to our study population. This justifies a placebo group as comparator. We assume that DXMT reduces postoperative opioid consumption. Not knowing the most effective regime justifies comparing one dose versus repeated doses of glucocorticoids.

In this trial, DXMT will be administrated in doses of 24mg being within the range of normally recommended doses for pain management, but beyond the recommended 4-10mg doses for PONV[13]. The trial medication (DXMT or placebo) will be administered intravenously starting just after induction of anaesthesia, and the following dose after 24 hrs.

7. Objectives and Hypothesis

Research hypothesis:

Perioperative administration of DXMT reduces postoperative morphine consumption compared to placebo after PAO in adults.

0-hypothesis

DXMT does not reduce postoperative morphine consumption compared to placebo after PAO in adults.

Primary Objective:

To compare the effect of intravenous DXMT relative to placebo on cumulated postoperative morphine consumption from baseline to 48 hours in hip dysplasia patients undergoing PAO.

Secondary Objective(s):

To compare the effect of repeated doses of intravenous DXMT relative to a single dose on cumulated postoperative morphine consumption from baseline to 48 hours in hip dysplasia patients undergoing PAO.

Moreover, to determine if DXMT is superior to placebo for:

- Perception of pain intensity postoperatively and need for supplemental analgesics
- Prevalence and degree of PONV, including antiemetic consumption
- Perception of sleep quality
- Mobilization postoperatively
- Prevalence and degree of serious adverse events (SAE), e.g. complicating wound infection, which need antibiotic treatment or revision surgery.

Explorative Objective:

To determine if DXMT relative to placebo:

- Affect the immune-response postoperatively
- Affect patient reported health and life quality, activity and pain

8. Study Design

The PAODEX trial is designed as a randomised, parallel group, placebo-controlled, superiority trial with three parallel groups. Treatment allocation will be performed with a block-size 3 to 6 with a 1:1:1 allocation.

STUDY TIMELINE (FLOW DIAGRAM)

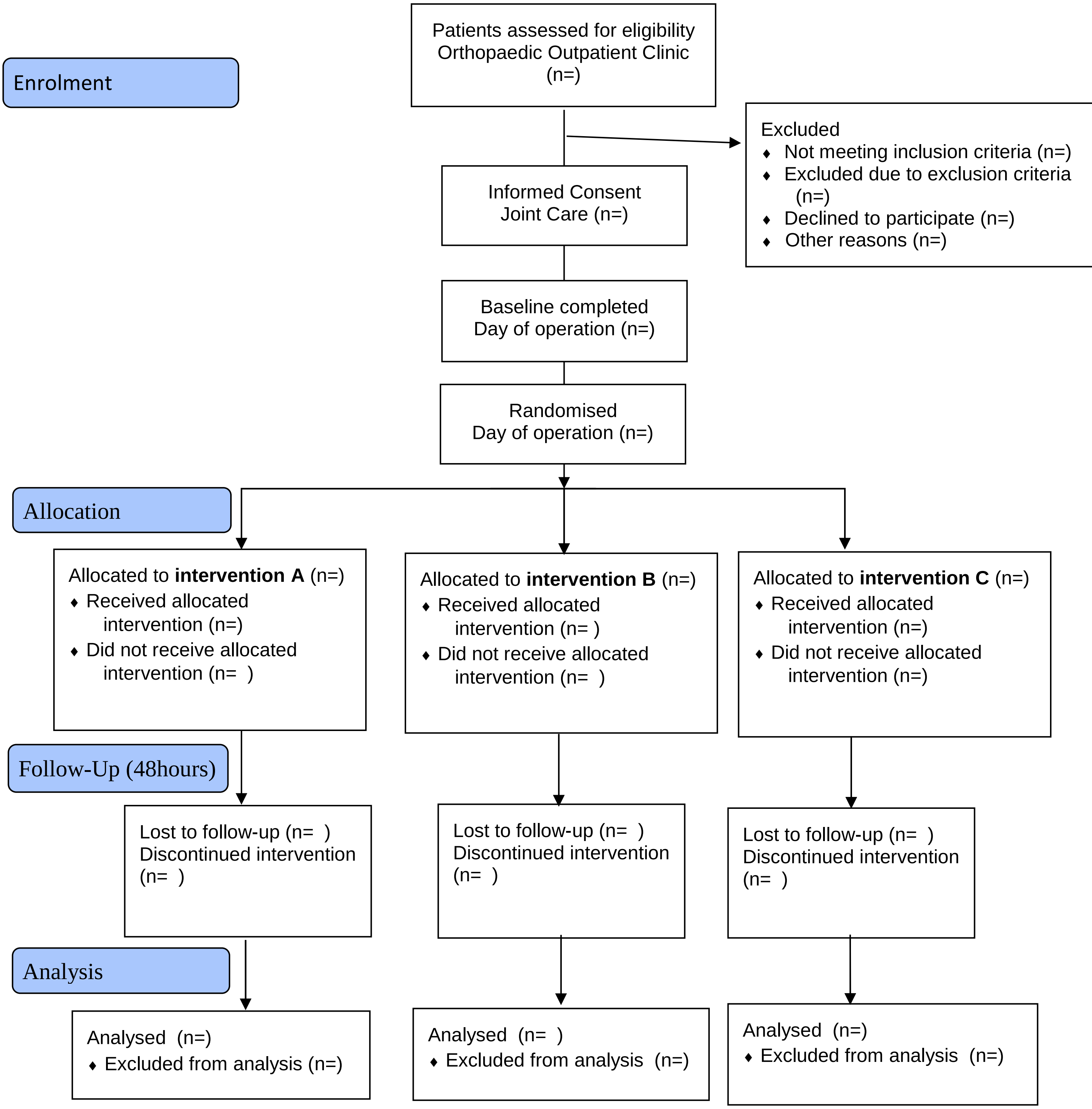

Table 1. Schedule for enrolment, interventions and assessments

		STUDY PERIOD										
	Enrolment	Allocation	Post-allocation									Close-out
TIMEPOINT	*JC* *-t_2*	*DOO* *-t_1*	*T_0*	*EOO* *t_1*	*3hrs* *t_2*	*24hrs* *t_3*	*48hrs* *t_4*	*POD14* *t_5*	*8w* *t_6*	*3mo* *t_7*	*6mo* *t_8*	*1yr t_9*
ENROLMENT:												
Eligibility screen	X											
Informed consent	X											
Baseline data	X											
Allocation		X										
INTERVENTIONS:												
Intervention period			◆—	—	—	—	—◆					
Trial Drug administration			X			X						
Follow-up							◆—	—	—	—	—	—◆
ASSESSMENTS:												
Sex, DOB, ASA-score, height, weight, blood pressure.	X											
Outcome measures*:						x	x	x	x	x	x	x
Other variables**;				x	x							X

JC; JointCare, DOO; day of operation, EOO; end of operation, POD; post operation day,
*Outcome Measures; details see data collection figure.
** Other variables; details see source data figure.

METHODS: PARTICIPANTS, INTERVENTIONS, OUTCOMES

9. STUDY SETTING

PAODEX is a single centre trial, conducted by the Department of Orthopaedic Surgery of Odense University Hospital in Denmark.

10. ELIGIBILITY CRITERIA

INCLUSION CRITERIA:

1. Patients undergoing PAO due to symptomatic hip dysplasia (CE<25grader) or retroverted acetabulum (crossover and posterior wall sign)
2. ≥ 18 years
3. Females if fertile*: Verified negative s-HCG, usage of safe contraceptives** or surgical sterilisation.
4. Patients who give their written informed consent to participating in the trial, after having fully understood the content of the protocol and restrictions.
   *Postmenopausal is defined as >12months amenorrhoea.
   **Safe contraceptives accepted: intrauterine device or hormone contraceptives.

EXCLUSION CRITERIA:

1. Patients who cannot speak or understand Danish
2. Allergy or contraindications to trial medication
3. Spinal anaesthesia
4. Second intervention carried out simultaneously (e.g. femur osteotomy)
5. Patients with daily opioid consumption prior to surgery (tramadol and codeine accepted)
6. Drug, medical abuse or weekly alcohol consumption beyond ≥7 (female) and ≥14 (men) units, respectively.
7. Mental disability, anxiety disorder (active psychiatric disorder or consumption of tricyclic antidepressants)
8. Diabetes diagnosed prior to inclusion
9. Immune suppression therapy (e.g. systemic glucocorticoids)
10. Kidney impairment (eGFR < 50ml/min) or liver disease (≥Child Pugh B)

11. INTERVENTIONS

INTERVENTIONAL DRUG:

<u>Dexamethasone</u>[31]:
Dispensing: 1ml ampules
Concentration: 4mg/ml,
Ancillary substances: Dinatriumedetat, Natriumcitrat (E 331), Natriumhydroxid (E 524), Water for injection.

Promoted in Denmark by:
Vital Pharma Nordic ApS, c/o Ordnung
Tuborgvej 5
DK 2900 Hellerup
Tel: 70276627
Fax: 69802829
Email: info@vitalpharmanordic.com

The Pharmaceutical manufacturer will be informed before initiation of trial.

PLACEBO DRUG:

Isotonic sodium-chloride[32]:
Dispensing: solvent for injection, no therapeutic impact.

DILUTIONAL DRUG:

Isotonic sodium-chloride[32]:
Dispensing: solvent for injection, no therapeutic impact.

Product summaries: Appendices 3 and 4.

11A INTERVENTIONS

Eligible patients will be randomized in equal proportions to one of three groups prior to surgery: Group A: Receive 24mg DXMT iv. after induction of anaesthesia (t_0) and again 24mg DXMT iv. after 24hrs. (t_3). Group B: receive 24mg DXMT iv. after induction of anaesthesia (t_0) and placebo iv. (isotonic saline) after 24 hrs. (t_3). Group C: receive placebo iv. both after induction of anaesthesia (t_0) and after 24 hrs. (t_3).

Group names	DOO (before surgery)	POD1, 24hrs after 1st dose
Group A	24mg DXMT iv	24mg DXMT iv
Group B	24mg DXMT iv	Placebo iv
Group C	Placebo iv	Placebo iv

Handling of trial drugs; see section 17 Blinding.

11B MODIFICATIONS

Reasons for discontinuing allocated interventions:
A patient can be withdrawn from the trial under following conditions:

- If withdrawal of consent: The participant can anytime withdraw his/her consent for participating the trial and the usage of achieved data.
- If the investigator believes that change of treatment will be best for the patient, including:

- Allergic reactions: Allergic reactions to DXMT is considered extremely rare (<0,01%)[33]. If this is suspected withdraw of trial medication from the participant. This should be reported as a serious adverse event (SAE).
- Biological disturbances: In general, biological disturbances related to DXMT are dependent on dose and term of treatment. A single high-dose glucocorticoid results in a transient (<48hrs) postoperative increase in plasma glucose in patients >55 years without insulin dependent diabetes after total joint arthroplasty[34].
- Hyperglycaemia after DXMT may reflect latent diabetes, and the condition is asymptomatic and reversible in healthy non-diabetic patients and sufficiently treated diabetics[35]. If symptomatic, the condition is treated medically, and the participant is withdrawn from the intervention. This will be reported as an adverse event. If possible, the participant remains in the trial to enable follow-up.
- Adrenal impairment is formerly investigated and is considered to be reversible and clinically irrelevant in short-term use[7,8], and will not be monitored further in this trial.

11C Adherence

Strategy to improve adherence during hospital stay:

- Before surgery, the participant will receive personal education by trial investigator or trial nurse to prepare, motivate and educate the participants to understand the importance of good quality of data to provide serious research.
- During the hospital stay; daily follow-up by investigator or trial nurse to support and motivate data registration and adherence, and to give the participant the opportunity to ask questions.
- Education of the participants and post anaesthesia care unit (PACU) personnel to fulfil the case-report-form (CRF).
- Education of ward staff to encourage participants to fill in their eCRF.

Strategy to improve adherence after discharge:

- We intend to use text messages to remind the participant to fill in the eCRF.
- Contact information to trial investigators in case of questions.

11D Concomitant Care

General set-up

Standard pre-medication: None.

Anaesthesia: General anaesthesia: Induction with propofol (10mg/ml) 2-4mg/kg followed by continuous administration of 5-15mg/kg/h, and remifentanil (50ug/ml) 1ug/kg followed by continuous administration of 0.5-1ug/kg/min. Both adjusted to clinical response and pharmacokinetics. Airway management with laryngeal mask. No neuromuscular blockade planned. Neutral perioperative fluid balance (perspiration; 2ml/kg/hrs), bleeding is substituted with crystalloid 1:2, larger bleeding, defined as >20% for estimated blood volume or haemoglobin <4.3mmol/l, is treated according to existing guidelines. Iv. fentanyl loading

during the entire surgery; initially 4ug/kg, additional supply of fentanyl 1-2ug/kg according to clinical response. Expected cumulated dose during surgery is 8-12ug/kg fentanyl.
Surgery: Standard guidelines for preparation, disinfection and sterile approach. Surgery is performed by 3 surgeons (SO, OO, MB) using the same modified Smith-Petersen approach and surgical technique[36]. No wound drains or local infiltration anaesthesia will be used.

Postoperative pain and nausea regime:

- In the recovery room:
  Morphine is administered after PACU guidelines. When the patient is sufficiently recovered from anaesthesia[37] and visual analog scale (VAS) <4, a patient-controlled-analgesia (PCA) pump with morphine bolus 0.04mg/kg, lock-out 8 minutes continued for 48 hours, if VAS > 3 a PCA bolus is recommended.
  Standard analgesics consist of tablet paracetamol 1g 6 hourly, tablet ibuprofen 400mg 6 hourly.
  Moderate to severe PONV is treated with 1st line: ondansetron titrated doses of 2 mg iv (maximum 8mg daily), 2nd line: Droperidol 0.625 -1.25mg iv. Administration by PACU personnel or ward nurses.

Standard non-pain or nausea medicine regime:

- Iv. dicloxacillin 2 grams, initiated at the operating theatre and administrated by the anaesthetic nurse, and continued with supplying 1 gram 8, 16, and 24 hours postoperatively.
- Intraoperative tranexamic acid 15mg/kg iv. (maximum 1 gram) to reduce bleeding during surgery. Administration by anaesthetic nurse.
- Thrombosis prophylaxis with low-molecular-weight heparin administered subcutaneously (5000IE Dalteparin) 24 hourly until POD7.
- Opioid constipation prophylaxis with Bisacodyl 10mg 24 hourly, administration by ward nurse or self-administration.

After ended intervention period (>48hrs):

- Patient will follow standard practise for analgesia of the Department of Orthopaedic Surgery.

Rescue medication:

It is of great importance that this is clearly registered in the CRF.
Pain: Tablet oxycodone 5 mg can only be considered in case of insufficient effect of the PCA pump.
Shivering: In case of persistent shivering, in spite of attempt to reheat, inj. pethidine 0.5mg/kg iv. administration is allowed.

Prohibited concomitant medication:

Analgesics: Other opioid agonists, pregabaline, gabapentin, clonidine and ketamine.
Antiemetic: Dexamethasone
Other medication: Any glucocorticoid, tricyclic antidepressants.

Listed drugs are expected to influence trial results.
All non-analgesic medications are permitted at the discretion of the attending doctor.

12. Outcomes

Primary Outcome:

Cumulated postoperative morphine consumption in milligrams after 48hours.

Key Secondary Outcomes:

- Postoperative pain intensity after 48 hrs. Evaluated at (*i*) rest and (*ii*) under the timed-up-and-go (TUG) procedure (Appendix 5). Pain intensity is assessed using the VAS.
- Cumulated consumption of opioids from 48hrs post operation to POD14.
- Postoperative nausea, 4point scale after 48hrs (Appendix 6).
- Cumulated antiemetic consumption in mg and drug will be assessed after 48hrs.
- TUG test after 48hrs. (Appendix 5).
- Number of patients with one or more SAEs, including wound infection treated with antibiotics or revision, within 8 weeks after surgery. Defined after the ICH-GCP-guidelines. Patients will be asked to accept a follow-up visit after 8 weeks, supplemented with a look in "Fælles Medicinkort / FMK".

Explorative (tertiary) Outcomes:

- All of the above assessed after 24 hours
- Immune response: C-reactive protein (CRP), leucocytes, the cytokines; interleukin (IL)-2, IL-6, IL-10, tumor necrosic factor (TNF) and pentraxin 3 (PTX3) at baseline, after 3, 24 and 48 hrs. After 72 hrs if participant is not yet discharged.
- Patient evaluated life quality, health, activity and pain, based on EQ-5D, UCLA and Oxford Hip (Appendix 8) preoperatively, after 3 and 6 months and 1 year follow up.

13. Participant Timeline

See appendix 9 for study plan.

Trial period:
From randomisation to 365 days postoperative, and data collecting ends.

Intervention period:
48 hrs from t1= end of surgery.

Time schedule:
Enrolment is estimated to a period of two years. The patients will be included consecutively. Screening for eligibility and enrolment will be executed along with consent for the PAO procedure.

Informed consent and baseline data will be collected along with Joint Care (JC), where patients are prepared for surgery. Allocation takes place at the day of surgery.

The planned interventions, procedures and follow-up are planned in relation to already implemented guidelines for admission and ambulatory follow-up or online registrations. Therefore, participation will not result in additional hospital visits.

14. Sample Size and Power Considerations

In the planning of the PAODEX Trial a small pilot study (10 patients) was launched for the clinicians to have some empirical data to support their clinical hypothesis (e.g. practical for power and sample size considerations). Two patients were exposed to perioperative DXMT administration (participants #9 and #10), whereas data was collected "as usual" from 8 patients (#1-#8; corresponding to placebo *per se*). Based on the sparse data available from the pilot study, basic descriptive statistics indicate that the overall mean is 47.47 mg morphine (over 48 hours), with a standard deviation (SD) of 32.69 mg morphine; these descriptive statistics together with the overall median (57.9 mg morphine) suggests that data might not be normally distributed however. Although questionable - because of the small sample sizes - the mean (SD) in the placebo and DXMT group corresponded to 56.8 (32.2) and 20.0 (14.1) mg morphine, respectively. We note that the poorly controlled – but potential clinical effect of adding DXMT – could correspond to an estimated treatment difference of 36.8 mg morphine *per se* (i.e. potential net benefit).

For a two-sample pooled t test of a normal mean difference with a two-sided significance level of 0.05, assuming a common standard deviation of 40 mg morphine, a total sample size of 87 patients assuming an allocation ratio of 2 to 1 (corresponding to group A&B vs group C) is required to obtain a power of at least 90% to detect an estimated treatment difference of 30 mg morphine. Anticipating some withdrawals and various minor protocol violations, it was decided to aim for enrolment of 90 participants in total (randomised: 30:30:30; i.e., $n_A + n_B = 60$ vs $n_C = 30$).

Based on the same assumptions as above, even if we are only able to enrol and randomise 66 patients in total (allocation ratio of 2 to 1; i.e. 44 vs 22 patients) we will obtain a reasonable statistical power of 80%.

According to our key secondary efficacy objective we also want to compare the effect of two doses (Group A) with a single dose (Group B) of intravenous DXMT: For a two-sample pooled t test of a normal mean difference with a two-sided significance level of 0.05, assuming a common standard deviation of 40 mg morphine, a total sample size of 60 patients assuming a balanced design ($n_A = n_B = 30$) has a statistical power of 81.5% to detect an estimated treatment difference of 30 mg morphine.

15. Recruitment

Screening of patients continues until target population is achieved. Yearly, about 100 patients undergo PAO at our centre, and we expect to reach an inclusion rate of 50%.

For details regarding implementation and consent, see section 26.

Methods: Assignment Of Interventions

16. Allocation

16a Sequence Generation

Participants will be randomised in a 1:1:1 manner to either control group (C) or one of two experimental groups (A or B); stratified randomisation will be based on gender (male vs. female).

A computer-generated randomisation sequence will be produced using Resource Electronic Data Capture (REDCap) to generate six randomisation sequences before any participant is enrolled, allocating participants in permuted blocks of sizes 3 to 6. The block sizes will not be disclosed to ensure concealment.

16b Concealment Mechanism

Participants giving consent and fulfilling the inclusion criteria will be randomly allocated based on the sequence generation provided above using REDCap an online randomisation service offered by Odense University Hospital and the Department of clinical Research University of Southern Denmark. Allocation concealment will be ensured until the day of surgery. OPEN data manager, who also allocates user rights to ensure blinding of the personnel performing the randomisation, makes the settings for REDCap.

16c Implementation

The orthopaedic surgeon finding indication for PAO performs enrolment of participants. An investigator or the trial nurse will use REDCap for executing the randomisation and allocation procedure. Primary investigator is major responsible for the randomisation process.

17. Blinding (masking)

17a Blinding (masking)

All interventions are blinded to the participants, the ones administering the intervention, researchers, other care providers, outcome assessors, as well as the statistician and investigators.

Above mentioned randomisation sequence will be entered into REDCap by OPEN data manager, who has no contact to the clinical trial, as well as REDCap will retain the non-blinded allocation list which is concealed by OPEN data manager until data has been collected, analysed and conclusions submitted.

Procedure for preparation of trial drug:

First dose DOO:

Intervention drug: 24mg DXMT (6ml of 4mg/ml) will be aspirated into a 10ml syringe. Sealed with a plug.

Placebo: 6ml sterile isotonic saline will be aspirated into a 10ml syringe. Sealed with a plug.

Second dose POD1:

Intervention drug: 24mg DXMT (6ml of 4mg/ml) will be aspirated into a syringe and added to a 100ml container with isotonic saline, concentration: 0,24mg/ml.

Placebo: 6ml sterile isotonic saline will be aspirated into a syringe and added to a 100ml container with isotonic saline. Both containers will be marked with expiration date and time, as the trial mixture containing DXMT is durable within 24 hours.

Delivering of intervention drug and placebo in similar containers is challenging and with expenses way beyond reasonable budget of this project. Active drug/placebo from the stock of the orthopaedic theatre at Odense University Hospital will be used. Anaesthetist personnel without any relation to the study will prepare the trial drug in accordance with good manufacture practice rules. After preparation, the syringe/container will be double controlled and labelled as trial drug. The person preparing the drug notes batch-no. and expiration time and date on the instruction paper and replace it into the 'randomisation envelope', which is sealed with a signed and dated label. The 'randomisation envelope' will be locked in a room where to investigators/study nurse have access.

A standard operating procedure for keeping an account of trial medication (incl. placebo and intervention drug) will be elaborated and stored at the preparation site and in the trial master file.

Administration:

First dose: The syringe with 6ml (active or placebo) will be delivered to the anaesthetic nurse at the operating theatre and is administered iv. after induction of anaesthesia. All personnel at the operating theatre will be blinded to the intervention and an investigator will be present.

Second dose: The container 106ml (active or placebo) will be delivered to the ward nurse at department O3, who will prepare an infusion line and administer the trial drug iv. over 10 minutes within the 24 hour expiration time. Therefore, administration will be 24 hours after

start of surgery as the latest. The choice of second dose administration aims to eliminate the sense of perineal pruritus potentially induced by iv. DXMT[39]. All personnel at the ward O3 will be blinded to the intervention and an investigator or study nurse will be present.

17B EMERGENCY UNBLINDING

Unblinding is permissible under specified circumstances.

If a SAE (according to the IHC-GCP definition, see section: Harms and adverse events) occurs during the intervention period (0-48 hours postoperatively) and the investigator, after consultation with either principal investigator, coordinating investigator or sponsor, finds it not feasible for the patient to continue, the trial medication will be discontinued. The participant will be asked whether to accept continued data recording including follow-up data.

The blinding may only be broken if the continued treatment of the patient requires knowledge of the allocation. The investigator can do this without restrictions.

Personnel without relation to the trial will prepare unblinding envelopes based on the non-blinded allocation list, delivered by OPEN data manager. The date and reason for unblinding must be recorded. Information about unblinding procedure is found in the trial master file. The trial master tile and unblinding envelopes are placed at 12th floor, ward O4, room 7, Odense University Hospital. The room is locked, but key is disposable day and night.

The investigator will ensure that necessary procedures and expertise to tackle an emergency situation that may arise during the trial are present.

METHODS: DATA COLLECTION, MANAGEMENT, ANALYSIS

18 DATA COLLECTION METHODS

18A DATA COLLECTION METHODS

Training:

Before inclusion, relevant personnel will be trained in study requirements, standardized measurement of height, weight, and blood pressure, requirements for laboratory specimen collection, counselling for adherence and the eliciting of information from study participants in a uniform reproducible manner.

Before trial, all participants will be introduced to using the eCRF by investigators or trial nurse. Participants will be taught how to report data quality.

Data will be collected in REDCap, a worldwide online system developed specifically for non-commercial clinical research.

Every patient included will have an eCRF, which will be signed by investigator to confirm data.

Corrections will be logged in REDCap and signed by investigator or a deputy. A paper CRF will follow the patient as long as admitted, after discharge the paper CRF will be deposited at Dept. of clinical research of Orthopaedic Surgery, Odense University Hospital.

Planned collection of source data:

Source data	Data	Course
Anaesthesia file and pre-operative evaluation	Height, weight, ASA-classification, blood pressure, sex, date of birth, smoking status, fluid administration, amount of bleeding, dispended anaesthetics and analgesics. Duration of operation.	For the use of comparison of groups after un-blinding
COSMIC, patient file system:<br>- admission history<br>- medicine module	Comorbidities<br>Medication administered from operation until discharge or 72 hrs. post operation	For the use of comparison of groups after un-blinding.<br>Analysing of primary and secondary outcome measures
Shared Medication Record (FMK)	Daily medicine consumption before operation	For the use of comparison of groups after un-blinding
COSMIC – integrated laboratory system and the extern system BCC-Web	CRP, Leucocytes, blood glucose, and standard analyses prior to PAO-procedure. Cytokines.	Analysing of explorative outcomes measures.<br>For the use of comparison of groups after un-blinding
Work sheet or eCRF (REDCap database)	PCA-pump morphine consumption.<br>Pain and nausea score. Timed up and go score, sleep score, patient reported outcome measures (see data collection figure)	Analysing primary, secondary and explorative outcome measures.

Following questionnaires or scales will be used in the trial:

- *Physical performance:* (Annex 8), Functionality; Oxford HIP, validated. Common wellbeing: EQ-5D, validated. Activity score: UCLA, validated.

Following scores will be used in the trial:

- The VAS[40,41] score is a scale ranging from 0 to 100 in which 0 indicates an absence of pain and 100 indicates unbearable pain. VAS score has formerly been used for scoring other types of outcomes e.g. sleep. VAS is a patient-reported outcome measure[40]. A difference between groups of at least 12mm will be considered a clinically relevant difference.
- VAS score in this study measures: Pain, validated, and sleep quality.
- Nausea intensity score: (appendix 6) 4 point is used; this is standard rating procedure in the PACU.

Figure: Data-collection (outcomes):

Variable	Before t_0	0hrs t_0	3hrs	24hrs	*48hrs	72hr	14d	8w	3mo	6mo	1yr	Time for data collection
**Baseline Characteristics**												
Sex	X											Before allocation
DOB	X											
ASA-score	X											
Height	X											
Weight	X											
Blood pressure	X											
**Blood Samples:**												
FBG	X		X	X	X	X						After ended follow-up
Inflammation	X		X	X	X	X						
**Medicine consumption:**												
Morphine				X	X		X					After 48 hrs, and POD 14.
Antiemetic				X	X							After 48 hrs
**Physical performance:**												
TUG				X	X							After 48 hrs
**PROM**												
Pain score				X	X							After 48 hrs
Nausea intensity score				X	X							After 48 hrs
Sleep	X			X	X							After 48 hrs
ED-Q5	X								X	X	X	After follow-up
Oxford-HIP	X								X	X	X	
UCLA	X								X	X	X	
**SAEs:**												
Patient-reported								X			X	After follow-up
Medical Record Review											X	After follow-up

*Primary endpoint assessment (48 hours from baseline)

DOB, Date of birth. ASA-score, American Society of Anaesthesiologists classification score. HbA1C, Haemoglobin A1C. FBG, fasting blood glucose. TUG, timed up and go. PROM, patient reported outcome measures. ISI, insomnia severity index. ED-Q5 EuroQol Group 5-dimention self-report questionnaire. UCLA, University of California, Los Angeles.

18B Retention

Primary outcome assessment is 48 hrs after ended surgery, where the participants are still hospitalized, as this is expected to maximize completeness of data collection. To make data collection after discharge more patient compliant, we intend to use online data registration and send reminders. Participants who do not complete data reporting will be contacted by investigators to elucidate the reason for dropout.

Procedure for patients who withdraw from the trial:

In accordance with the Declaration of Helsinki, patients have the right to withdraw from the trial at any time for any reason. The investigators also have the right to withdraw a patient from the trial at any time.

The reason and time for withdrawal before the scheduled time must be recorded in the patient's CRF. The participant will be asked if the withdrawal only applies the intervention such as further data registration is accepted OR if the withdrawal applies for any further intervention and data registration.

New participants will not replace participants who withdraw from trial after allocation.

Participants who withdraw from the trial during the intervention period will be analgised after existing guideline for pain and nausea management in the Department of Orthopaedic Surgery, Odense University Hospital. After discharge, management of pain and nausea will follow the existing guideline in the department.

19 Data Management

Entered data will be stored on OPEN's server in the Region of Southern Denmark. Data is entered via an encrypted connection and fulfil the demands for data security. All data entries and changes are logged in REDCap; therefore, the database may store e.g. social security number and also meets the GCP requirements for use of eCRF, when conducting medical trials. After ended study (last visit), all data will be pseudonymised.

20 Statistical Methods

20A Primary analyses

Analysis Populations

The primary analysis population will be based on the intention-to-treat (ITT) principle. In general, it is advantageous to demonstrate a lack of sensitivity of the principal trial results to alternatives of the set of participants analysed. We plan to conduct both an analysis of the full analysis set and a per protocol analysis, so that any differences between them can be explicitly discussed and interpreted. The primary analysis population will be based on the ITT principle. Thus, all data regardless of dropout and participation rate will be included as data in this analysis. The ITT principle asserts the effect of a treatment policy (that is, the planned treatment regimen) rather than the actual treatment given. It has the consequence that participants allocated to a treatment group should be followed up, assessed and analysed as members of that group irrespective of their adherence to the planned course of treatment.

The intention-to-treat population will include all randomised patients, regardless of their eligibility, according to the treatment they were randomised to receive.

The primary null hypothesis is based on the comparison between participants randomised to group A&B vs C; H0: $\mu_{A+B} = \mu_C$. The overall study design is based on three groups (A, B, and C); we apply linear modelling based on general linear models (GLMs) in the analysis of the primary and secondary outcome measures (continuous outcome measures): Analysis of covariance (ANCOVA) models will be performed, which produces several diagnostic measures, provides contrasts and estimates for customised hypothesis tests, and it provides tests for means adjusted for covariates (e.g., least squares [LS] means adjusted for baseline variables). The GLM procedure handles models relating a continuous dependent variable to one or several independent variables. Categorical outcomes for dichotomous endpoints will be analysed with the use of logistic regression with the same fixed effects and covariates as the respective analysis of covariance; for ease of interpretation the resulting Odds Ratios will subsequently be converted into Risk Differences with 95% Confidence Intervals (CI).

The independent variables can be either classification variables, which divide the observations into discrete groups (e.g., A, B, and C), or continuous variables (e.g., the level at baseline). We will analyse continuous outcomes using ANCOVA models, with a fixed factor for group (either 2 levels [A&B pooled vs C] and 3 levels [A vs B vs C], respectively) and adjust for the value at baseline. For each continuous outcome variable (after performing the overall ANCOVA model), we will obtain the P value and group contrast for the difference between the LS means: A vs. B (in analogy to a two-sample t test), independent of what the overall ANCOVA model indicates. Also, results following the ANCOVA model will be expressed as estimates of the group difference in the various pairwise comparisons in the changes from baseline, with 95% CIs to represent the precision of the estimates.

To assess the adequacy of the linear models describing the observed data, as well as to check the assumptions for the systematic and random parts of the models, we will investigate the model features via the predicted values and the residuals; that is, the residuals have to be normally distributed (around 0) and be independent of the predicted values.

All reported P values and Confidence Intervals will be two-sided and will not be adjusted for multiple comparisons per default (despite having 4 potential tests for each outcome variable $[3 \times \{3 - 1\}/2]$ plus the overall ANCOVA). Per default, we set the statistical significance at the conventional level of 0.05 ($P < 0.05$). All analyses will be performed using commercially available statistical software (e.g. SAS or STATA). An explicit Statistical Analysis Plan will be developed before trial closure (i.e. before breaking the blind).

20B ADDITIONAL ANALYSES
The final statistical plan will be described in the published manuscript.

20C MISSING DATA AND REPORTING OF PROTOCOL DEVIATIONS

Statistical methods to handle missing data: Loss to follow-up (and missing data for various reasons) is hard to avoid in randomised trials. We will apply the analysis framework suggested by White et al [42] for “ITT analyses” that depends on making plausible assumptions about the missing data and including all participants in sensitivity analyses:

1. Attempt to follow up all randomised participants, even if they withdraw from allocated treatment

2. Perform a main analysis of all observed data that are valid under a plausible assumption about the missing data

3. Perform sensitivity analyses to explore the effect of departures from the assumption made in the main analysis

4. Account for all randomised participants, at least in the sensitivity analyses.

The evaluability assessment of each participant in the statistical analyses will be performed before the code is revealed. Excluded participants and missing, unused or false data will be described.

Protocol deviations will be reported (defined as a failure to adhere to the protocol such as the wrong intervention being administered, incorrect data being collected and documented, errors in applying inclusion/exclusion criteria or missed follow-up visits). These will be defined as major or minor.

Protocol deviations will be defined prior to unblinding of data to avoid any bias being caused and due consideration given to inclusion of participants within analysis populations.

Explicit data analysis aspect (incl. compuattional syntax) and the observed protocol deviations may be reported in another document - the Statistical Analysis Plan (SAP); the SAP will be written and confirmed with closure as a pdf with date prior to unblinding of data to avoid any bias being caused and due consideration given to inclusion of participants within analysis populations.

METHODS: MONITORING

21 DATA MONITORING

21A FORMAL COMMITTEE

A data monitoring committee via the local Good Clinical Practice Unit (GCP) has been established to monitor data. The GCP is independent of the study organizers and ensure that the data collected is valid, complete, and well-documented. This with the purpose to ensure that the trial subject's rights, safety, and well-being are protected as described in the Declaration of Helsinki.

21B INTERIM ANALYSIS

No interim analysis is planned.

22 HARMS - ADVERSE EVENTS

Adverse events (AE) are defined as in the European Directives[43] and GCP[44].

AE definition: Side effects are defined as any adverse event, sign or symptom that occur during participation in the trial which are time related to the administration of the trial medication, whether the adverse events is considered to be related to the trial medication or not.

Adverse reaction (AR) definition: all untoward and unintended response to a medical product related to any dose administered.

SAE or Serious adverse reactions (SAR) definition: An event defined as any incident involving a significant risk of death or disability of the participant, including, but not limited to, an event that: results in death; is life-threatening – in the investigator’s opinion the participant was in immediate risk of death from the adverse event when it appeared; requires or prolongs hospitalisation; is permanently disabling or is a congenital anomaly.

Following will be considered predictable events related to surgery and general anaesthesia, and will therefore not be registered as AEs:

- Laboratory values outside normal range not requiring treatment
- Moderate hypotension (MAP <60 mmHg)
- “Shivering”
- Pain from the surgical field
- Urine retention
- Intraoperative bleeding
- Nausea and vomiting
- Dizziness
- Fatigue

All SAE will be recorded in the participants CRF from time of injection of trial drug until 168 hrs postoperative (POD7) (5x biological half-life[45]).

SAEs occurring after a participant is discontinued from the study will NOT be reported unless the investigators think that the event may have been caused by the study drug. Investigators will determine relatedness of an event to study drug based on a temporal relationship to the study drug, as well as whether the event is unexpected or unexplained given the participant's clinical course, previous medical conditions, and concomitant medications.

Patients with SAE will be monitored with appropriate clinical assessments and laboratory tests according to the decision of the attending doctor. All SAEs will be followed until satisfactory recovery or stabilization.

Reporting of SAE's:

The investigators are responsible for the ensuring that all SAE's are recorded in the patient's CRF.

The sponsor is responsible for continuous monitoring of the trial's risk/benefit relation. If situations arise that may affect the safety of the trial participants, or affect the performance of the trial, these must always be immediately reported to the Danish Medicines Agency. Similarly, reports should also be made to all investigators and Research Ethics Committees involved.

In addition, the following rules apply for reporting to the authorities. Events and reactions should be included for all trial medication – including the experimental treatment, the comparative treatment and any placebo.

SAE's should be reported immediately by the investigator to the sponsor (sponsor-investigator), within 24 hours.

(SAR) or serious presumed side effects - should be reported immediately by the investigator to the sponsor (sponsor-investigator), within 24 hours. SAR shall be reported by the sponsor (sponsor-investigator) annually to the Danish Medicines Agency and the Research Ethics Committee. The report shall also include a report on the safety of trial participants.

Suspected unexpected serious adverse reaction (SUSAR), unexpected and serious side effects - must be immediately reported by the sponsor (sponsor-investigator) to the Danish Medicines Agency and the Research Ethics Committee.

Fatal or life-threatening SUSARs must be reported within 7 days of the sponsor becoming aware of them, and within 8 days of the report the sponsor must inform the Danish Medicines Agency of all relevant information on the follow-up.

The summary of product characteristics: section 4.8, will be used as reference document in evaluation of expected SAR/SUSAR[31].

All other unexpected SUSARs should be reported to the same authorities within 15 days of the sponsor (sponsor-investigators) being informed of these.

All reports will be accompanied by comments on any consequences for the trial.

The sponsor shall at all time be kept informed by the investigators of any SAE's.

Once a year a report containing SAE's will be elaborated and send to The Regional Committees on Health Research Ethics for Southern Denmark and Danish Medicine Agency. A final report containing a description of all side effects will be elaborated after ended trial. Sponsor is responsible for this procedure, delegating this task to an investigator is accepted.

23 Auditing and quality assurance

Accepted procedure for quality control is done according to the ICH-guideline for Good Clinical Practice. Trial will be conducted in accordance with the existing protocol and statues in force and public authority requirements.

The local GCP unit at Odense University Hospital will have control of the statutory GCP-monitoring of the trial process. (see section 29 for access to data).

ETHICS AND DISSEMINATION

24 Research Ethics Approval

Approval by the Regional Committees on Health Research Ethics for Southern Denmark: S-20190017.

24A Ethical considerations

A single dose DXMT iv. is commonly used as an analgesic treatment and PONV prophylaxis as described in background section. The optimal dose, regimen and combination with other analgesics remains to be established. Also, higher and consecutive doses have not been thoroughly investigated for its pain alleviating effects in orthopaedic procedures, and to our knowledge, never investigated for the PAO population. We find it particularly important to study the analgesic effect of DXMT in patients undergoing PAO, as these patients often suffer from severe postoperative pain and PONV, which has been found difficult to treat.

Participation in the trial gives participants the opportunity to administer the additional analgesic treatment with morphine themselves. Self-administration of opioids has been shown to give a high level of participant satisfaction. Apart from adjusting pain treatment themselves, participants will not necessarily benefit directly from the trial participation – beside benefits from closer observation. However, it is expected that the information gathered will lead to a potential sizable improvement in pain and PONV management after PAO in the future, including better mobilisation and rehabilitation postoperatively.

Participation is considered ethically acceptable by the project group.

25. Protocol Amendments

If important protocol modifications are added subsequently, relevant parties (e.g. participants, investigators, trial registries) will be informed.

26. Consent

All participants considered for the trial will be provided with written and oral trial information enabling the participants to make an informed decision about participation in the trial.

Written information and the consent form will be subjected to review and approval by the Committees on Health Research ethics committee (appendix 10 and 11). This consent form must be signed by the participant and by the investigator or trial nurse.

Screening:
All patients scheduled for PAO at the orthopaedic outpatient department at OUH, will be asked permission for screening for implementation in the trial by the surgeons SO, OO and MB. The surgeons will complete a screening form determining patient eligibility and inform eligible patients that the screening form is handed to the investigators.

Patient information:
Eligible patients will be adequate verbally information about aim of study, study course, expected benefits and potential risks and rights as trial participants. Written information will be handed out (appendix 10). The patients will be informed of the possibility of having a companion present. The interview will be conducted in a closed room without distractions or interruptions. The patients will be given relevant and necessary time to consider the request, minimum 2 days. The patients will also be informed that participation is voluntary and that they may withdraw from the trial at any time. Patients will be informed that they will receive standard care after current practice if they choose not to participate or wish to withdraw from the trial. Patients will be informed that randomisation will take place on the day of surgery.

Furthermore, the patients will be informed that all information obtained during the trial will be treated with strict confidentiality and anonymised. And that The Danish Data Protection Agency and The Danish Medicines Agency will supervise the project. They are also informed that in regard to national law The Danish Medicines Agency together with sponsor, investigator and the GCP-unit will have access to the participant's journal in order to perform control and inspection of the project. And that all parties above are bound to secrecy.

Informant:
The orthopaedic surgeon SO, OO, MB or study nurse AGP will give verbal as written information, and obtain the patients consent at Joint Care (the day for preparation to surgery) about 7-14 days before surgery. A copy of the information and consent declaration will be given to the participants. (Appendix 10 and 11).

Informed consent:
Can be obtained when the patient is written and verbally informed, has been given relevant time to consider the request, and it is ensured that all relevant information is understood. This includes that the consent gives sponsor and his deputies and monitor access to patient journals, to assure data relevant for the trial, as well as the consent gives access to relevant authorities in relation to auditing (see section 23).

Informed consent shall be obtained on the day of surgery at the latest.

27. CONFIDENTIALITY

All study-related information will be stored securely at the study site. All participant information will be stored in locked cabinets in areas with limited access.
Participant information will be protected by the Rules on Protection of Personal Data and the Danish Health Act. The PAODEX trial will be reported to the Danish Data Protection Agency.

All laboratory results, data collection, administrative forms will be identified by coded ID to maintain participant confidentiality (pseudonymised). All records containing names or other personal identification as informed consent will be stored separately from study records identified with code number.
All local databases will be secured with password-protected access systems.

28. Declaration of Interests

None of the members of the trial group have any conflicts of interests in relation to present study.
The GCP-unit monitoring the trial on behalf of sponsor has no conflicts of interests.

29. Access to Data

The investigators will have access to the full trial data set and are responsible for managing and archiving data in accordance with current regulations. Projected data will be housed on REDCap via OPEN and all data sets will be password-protected.

Data collected in eCRF and records will only be made available to third parties in accordance with Danish law; during monitoring by Odense University Hospital GCP unit, as well as upon inspection, by authorized representatives of the relevant authorities.

30. Ancillary and Post-Trail Care

No financial provision to the participants enrolled in the trial is planned. Participants enrolled into the study are covered by indemnity for negligent harm by a publicly funded compensation scheme, which covers all areas of the Danish Health Care system. The scheme covers, if the trial subject is injured in connection with treatment, including medicinal product injuries.

31. Dissemination Policy

31a Trial Results and Dissemination Policy

Release of results:

All presentations are expected to protect the integrity of the major objectives of the study. End-point data must be presented by the Project Group.

Review process:

Each paper or abstract extracted from this trial must be submitted to the Project Group for review of its appropriateness and scientific merit prior to submission, the committee may recommend changes to the authors. Approval from the Project Group must be permitted before submission for publication.

On the basis of the data, the investigators will write a report of the trial. This report will be forwarded to relevant authorities. The report will also form the basis of manuscripts to be submitted for publication in international journals and presented at relevant meetings. Both positive, negative and inconclusive results will be submitted for publication.

31b Authorship

Authorship is assigned according to the ICMJE recommendations (Vancouver recommendations) based on 4 criteria[46].

Manuscripts outgoing from primary and secondary outcome results of this trial to submission for publication, goes with the following order of authors: *author sequence will be revised and may be changed in accordance to contribution*.

1. Viktoria Lindberg-Larsen
2. Stine T. Zwisler
3. Stine Hebsgaard
4. Ole Ovesen
5. Peter Lindholm
6. Morten Bøgehøj
7. Robin Christensen
8. Søren Overgaard

For following explorative reports, authorships will be determined for each report assigned according to ICMJE recommendations.

There is no intended use of professional writers. Both negative and positive results will be published. Proofreading is intended.

31c Reproducible Research

In order to enhance transparency and reproducibility the project group intend to publish the trial protocol.

APPENDICES

32. INFORMED CONSENT MATERIALS

Appendix no 10: Trial information, participant. Danish.
Appendix no 11: Consent declaration, Danish.

32A OTHER DOCUMENTS

Appendix no 1: Planned Budget
Appendix no 2: Protocol Summary for lay people, Danish
Appendix no 3: Summary of product characteristics of dexamethasone, Danish
Appendix no 4: Summary of product characteristics of isotonic saline, Danish
Appendix no 5: Timed Up & Go procedure, Danish
Appendix no 6-7: Nausea score and VAS score, Danish
Appendix no 8: PROM Questionnaires: Oxford Hip, ED-5Q, UCLA, Danish
Appendix no 9: Trial plan
Appendix no 12: Abbreviation list

33. BIOLOGICAL SPECIMENS

We intend to collect *blood samples* for storage in a biobank by indication of laboratory analyses on an accumulated basis. We intend to collect samples for specified cytokine analyses (see data-collection figure).

We intend to collect 60 ml blood per participant, a few participants 74 ml (this applies for those admitted after 72 hours)

Analysing is planned on an accumulated basis after last patient is discharged. Extra material will be destroyed. We have no intension of storage for later unspecified analyses. After ended trial the data will be pseudonymised and saved for 5 years under current legislation. Extra material will not be handed over to a third part. We have no intention of the materials goes out of the country.

Application for approval for storage in a biobank by the regional Danish Data Protection Agency is planned.

34. References:

1. Chan A-W, Tetzlaff JM, Gotzsche PC, et al. SPIRIT 2013 explanation and elaboration: guidance for protocols of clinical trials. *BMJ*. 2013;346(jan08 15):e7586-e7586. doi:10.1136/bmj.e7586
2. Moher D, Hopewell S, Schulz KF, et al. CONSORT 2010 explanation and elaboration: Updated guidelines for reporting parallel group randomised trials. *Int J Surg*. 2012;10(1):28-55. doi:10.1016/j.ijsu.2011.10.001
3. Kehlet H, Dahl JB. Anaesthesia, surgery, and challenges in postoperative recovery. *Lancet (London, England)*. 2003;362(9399):1921-1928. doi:10.1016/S0140-6736(03)14966-5
4. Morfin "Alternova" https://pro.medicin.dk/Medicin/Praeparater/7624. Accessed February 22, 2018.
5. Zaltz I, Beaulé P, Clohisy J, et al. Incidence of Deep Vein Thrombosis and Pulmonary Embolus Following Periacetabular Osteotomy. doi:10.2106/JBJS.J.01769
6. Salerno A, Hermann R. Efficacy and safety of steroid use for postoperative pain relief. Update and review of the medical literature. *J Bone Joint Surg Am*. 2006;88(6):1361-1372. doi:10.2106/JBJS.D.03018
7. Williamson LW, Lorson EL, Osbon DB. Hypothalamic-pituitary-adrenal suppression after short-term dexamethasone therapy for oral surgical procedures. *J Oral Surg*. 1980;38(1):20-28.
8. Spiegel RJ, Vigersky RA, Oliff AI, Echelberger CK, Bruton J, Poplack DG. Adrenal suppression after short-term corticosteroid therapy. *Lancet (London, England)*. 1979;1(8117):630-633.
9. Smith C, Erasmus P, Myburgh K. Endocrine and Immune Effects of Dexamethasone in Unilateral Total Knee Replacement. *J Int Med Res*. 2006;34:603-611.
10. Backes JR, Bentley JC, Politi JR, Chambers BT. Dexamethasone Reduces Length of Hospitalization and Improves Postoperative Pain and Nausea After Total Joint Arthroplasty: A Prospective, Randomized Controlled Trial. *J Arthroplasty*. 2013;28(8):11-17. doi:10.1016/J.ARTH.2013.05.041
11. LUNN TH, KEHLET H. Perioperative glucocorticoids in hip and knee surgery - benefit vs. harm? A review of randomized clinical trials. *Acta Anaesthesiol Scand*. 2013;57(7):823-834. doi:10.1111/aas.12115
12. Richardson AB, Bala A, Wellman SS, Attarian DE, Bolognesi MP, Grant SA. Perioperative Dexamethasone Administration Does Not Increase the Incidence of Postoperative Infection in Total Hip and Knee Arthroplasty: A Retrospective Analysis. *J Arthroplasty*. 2016;31(8):1784-1787. doi:10.1016/j.arth.2016.01.028
13. Chen P, Li X, Sang L, Huang J. Perioperative intravenous glucocorticoids can decrease postoperative nausea and vomiting and pain in total joint arthroplasty: A meta-analysis and trial sequence analysis. *Medicine (Baltimore)*. 2017;96(13):e6382. doi:10.1097/MD.0000000000006382
14. Nurok M, Cheng J, Romeo GR, Vecino SM, Fields KG, YaDeau JT. Dexamethasone and perioperative blood glucose in patients undergoing total joint arthroplasty: A retrospective study. *J Clin Anesth*. 2017;37:116-122. doi:10.1016/j.jclinane.2016.11.012
15. Hartman J, Khanna V, Habib A, Farrokhyar F, Memon M, Adili A. Perioperative systemic glucocorticoids in total hip and knee arthroplasty: A systematic review of outcomes. *J Orthop*. 2017;14(2):294-301. doi:10.1016/j.jor.2017.03.012
16. Toner AJ, Ganeshanathan V, Chan MT, Ho KM, Corcoran TB. Safety of Perioperative

Glucocorticoids in Elective Noncardiac Surgery: A Systematic Review and Meta-analysis. *Anesthesiology*. 2017;126(2). doi:10.1097/ALN.0000000000001466
17. Jørgensen CC, Pitter FT, Kehlet H. Safety aspects of preoperative high-dose glucocorticoid in primary total knee replacement. *Br J Anaesth*. 2017;119(2). doi:10.1093/bja/aex190
18. Kamath AF. Bernese periacetabular osteotomy for hip dysplasia: Surgical technique and indications. *World J Orthop*. 2016;7(5):280-286. doi:10.5312/wjo.v7.i5.280
19. Clohisy JC, Schutz AL, St John L, Schoenecker PL, Wright RW. Periacetabular osteotomy: a systematic literature review. *Clin Orthop Relat Res*. 2009;467(8):2041-2052. doi:10.1007/s11999-009-0842-6
20. Jacobsen S, Sonne-Holm S, Lund B, et al. Pelvic orientation and assessment of hip dysplasia in adults. *Acta Orthop Scand*. 2004;75(6):721-729. doi:10.1080/00016470410004094
21. Sankar WN, Duncan ST, Baca GR, et al. Descriptive epidemiology of acetabular dysplasia: The Academic Network of Conservational Hip Outcomes Research (ANCHOR) periacetabular osteotomy. *J Am Acad Orthop Surg*. 2017;25(2):150-159. doi:10.5435/JAAOS-D-16-00075
22. Jacobsen JS, Nielsen DB, Sørensen H, Søballe K, Mechlenburg I. Changes in walking and running in patients with hip dysplasia. *Acta Orthop*. 2013;84(3):265-270. doi:10.3109/17453674.2013.792030
23. Clohisy JC, Ackerman J, Baca G, et al. Patient-Reported Outcomes of Periacetabular Osteotomy from the Prospective ANCHOR Cohort Study. *J Bone Joint Surg Am*. 2017;99(1):33-41. doi:10.2106/JBJS.15.00798
24. Lindberg-Larsen V, Bandholm TQ, Zilmer CK, Bagger J, Hornsleth M, Kehlet H. Preoperative methylprednisolone does not reduce loss of knee-extension strength after total knee arthroplastyA randomized, double-blind, placebo-controlled trial of 61 patients. *Acta Orthop*. 2017;88(5):543-549. doi:10.1080/17453674.2017.1345236
25. Belmont PJ, Goodman GP, Waterman BR, Bader JO, Schoenfeld AJ. Thirty-Day Postoperative Complications and Mortality Following Total Knee Arthroplasty. *J Bone Jt Surgery-American Vol*. 2014;96(1):20-26. doi:10.2106/JBJS.M.00018
26. Namba RS, Inacio MCS, Paxton EW. Risk factors associated with surgical site infection in 30 491 primary total hip replacements. *J Bone Joint Surg Br*. 2012;94-B(10):1330-1338. doi:10.1302/0301-620X.94B10.29184
27. Weilbach C, Rahe-meyer N, Raymondos K, Weissig A, Scheinichen D, Piepenbrock S. Postoperative nausea and vomiting (PONV) : usefulness of the Apfel-score for identification of high risk patients for PONV. *Acta Anaesthesiol Belg*. 2006;57(4):361-363.
28. Bech RD, Ovesen O, Lindholm P, Overgaard S. Local anesthetic wound infiltration for pain management after periacetabular osteotomy. A randomized, placebo-controlled, double-blind clinical trial with 53 patients. *Acta Orthop*. 2014;85(2):141-146. doi:10.3109/17453674.2014.899840
29. Salerno A, Hermann R. Efficacy and Safety of Steroid Use for Postoperative Pain Relief. *J Bone Jt Surg*. 2006;88(6):1361-1372. doi:10.2106/JBJS.D.03018
30. Danish Medicines Agency. Dexavital. https://pro.medicin.dk/Medicin/Praeparater/8720. Accessed May 23, 2018.
31. Danish Medicine Agency. Dexavital. 27. October. http://produktresume.dk/AppBuilder/search?button=Søg&expand_all=false&id=&page=0&q=dexamethason+AND+%22injektionsvæske%22&refinements_token=%7B%7D&selected_tokens%5B%5D=%7B%22s%22%3A%5B%7B%22id%22%3A%22folder-

refinement%22%2C%22xPath%22%3A%22%24. Published 2017. Accessed May 31, 2018.
32. Danish Medicine Agency. Natriumchlorid 9mg/ml. 15. august. http://produktresume.dk/AppBuilder/search?utf8=✓&id=&type=&q=natriumklorid&button=Søg. Published 2015. Accessed May 31, 2018.
33. PRODUKTRESUMÉ. for. Solu-Medrol, pulver og solvens til injektionsvæske, opløsning - PDF. http://docplayer.dk/18288752-Produktresume-for-solu-medrol-pulver-og-solvens-til-injektionsvaeske-oploesning.html. Accessed February 19, 2018.
34. Lindberg-Larsen V, Kehlet H, Bagger J, Madsbad S. Preoperative High-Dose Methylprednisolone and Glycemic Control Early After Total Hip and Knee Arthroplasty. *Anesth Analg*. June 2018:1. doi:10.1213/ANE.0000000000003591
35. Hans P, Vanthuyne A, Dewandre PY, Brichant JF, Bonhomme V. Blood glucose concentration profile after 10 mg dexamethasone in non-diabetic and type 2 diabetic patients undergoing abdominal surgery. *Br J Anaesth*. 2006;97(2):164-170. doi:10.1093/bja/ael111
36. Hussell JG, Mast JW, Mayo KA, Howie DW, Ganz R. A comparison of different surgical approaches for the periacetabular osteotomy. *Clin Orthop Relat Res*. 1999;(363):64-72.
37. Jepsen CH. No Title. OUH, guideline, Dokument ID 73222.
38. Smith MT, Wegener ST. Measures of Sleep The Insomnia Severity Index, Medical Outcomes Study (MOS) Sleep Scale, Pittsburgh Sleep Diary (PSD), and Pittsburgh Sleep Quality Index (PSQI) INSOMNIA SEVERITY INDEX General Description. doi:10.1002/art.11409
39. Perron G, Dolbec P, Germain J, Béchard P. Perineal pruritus after iv dexamethasone administration. *Can J Anesth Can d'anesthésie*. 2003;50(7):749-750. doi:10.1007/BF03018722
40. Breivik H, Borchgrevink PC, Allen SM, et al. Assessment of pain. *Br J Anaesth*. 2008;101(1):17-24. doi:10.1093/bja/aen103
41. Hartrick CT, Kovan JP, Shapiro S. The numeric rating scale for clinical pain measurement: a ratio measure? *Pain Pract*. 2003;3(4):310-316. doi:10.1111/j.1530-7085.2003.03034.x
42. White IR, Horton NJ, Carpenter J, Pocock SJ, Pocock SJ. Strategy for intention to treat analysis in randomised trials with missing outcome data. *BMJ*. 2011;342(feb07 1):d40. doi:10.1136/bmj.d40
43. Medicines Agency E. Guideline on the clinical development of medicinal products intended for the treatment of pain Adoption by CHMP for release for 2 Guideline on the clinical development of medicinal products intended for the treatment of pain. 2016.
44. Guideline for good clinical practice E6(R2). 1995.
45. McKee MD. Efficacy and safety of steroid use for postoperative pain relief. *J Bone Joint Surg Am*. 2007;89(5):1134; author reply 1134.
46. ICMJE | Recommendations | Defining the Role of Authors and Contributors. http://www.icmje.org/recommendations/browse/roles-and-responsibilities/defining-the-role-of-authors-and-contributors.html. Accessed February 21, 2018.
