# Supplementary material for: Effect of high-dose dexamethasone on morphine use after periacetabular osteotomy for hip dysplasia: a randomized double-blind placebo-controlled single center trial
Source: Acta Orthop. 2025 Jun 1;96:413–20. doi: 10.2340/17453674.2025.43903 (PMC12128623; doi:10.2340/17453674.2025.43903)
Supplement: Supplementary file 2 [file ActaO-96-43903-s2.pdf]

# Statistical Analysis Plan for the PAODEX Trial

## The effect of perioperative dexamethasone administration on postoperative pain in patients undergoing periacetabular osteotomy: A randomised placebo-controlled trial

March 25st, 2024

---

Viktoria Lindberg-Larsen[1,2], Ole Ovesen[3], Stine Thorhauge Zwisler[1], Peter Lindholm[1], Stine Hebsgaard[1], Martin Lindberg-Larsen[3,4], Robin Christensen[2,5], Søren Overgaard[3,6,7]

**Affiliations:**
[1]Department of Anaesthesiology and Intensive Care Medicine, Odense University Hospital, Odense, Denmark
[2]Section for Biostatistics and Evidence-Based Research, The Parker Institute, Bispebjerg and Frederiksberg Hospital, Copenhagen, Denmark
[3]Department of Orthopaedic Surgery and Traumatology, Odense University Hospital, Odense, Denmark
[4]Orthopaedic Research Unit, Department of Clinical Research, University of Southern Denmark, Odense University Hospital, Odense, Denmark
[5]Research Unit of Rheumatology, Department of Clinical Research, University of Southern Denmark, Odense University Hospital, Odense, Denmark
[6]Department of Orthopaedic Surgery and Traumatology, Copenhagen University Hospital, Bispebjerg Hospital, Copenhagen, Denmark
[7]Department of Clinical Medicine, Faculty of Health and Medical Science, University of Copenhagen, Copenhagen, Denmark

**Primary Investigator:** Viktoria Lindberg-Larsen (VLL), Department of Anaesthesiology and Intensive Care Medicine, Odense University Hospital, Odense, Denmark

**Senior Biostatistician and Statistical Analyst:** Robin Christensen (RC), Professor of Biostatistics and Clinical Epidemiology: Section for Biostatistics and Evidence-Based Research, The Parker institute, Bispebjerg and Frederiksberg Hospital, Copenhagen, Denmark and Research Unit of Rheumatology, Department of Clinical Research, University of Southern Denmark, Odense University Hospital, Odense, Denmark

**Database Manager:** Thon Kowal Andersen (TKA), Open Patient data Explorative Network (OPEN), Odense University Hospital, Region of Southern Denmark

**Trial Registration Number:** ClinicalTrials.gov (NCT03874936)

**Protocol version:** This document is written based on the information contained in the trial protocol version 4.0, dated May 7th 2021.

## INTRODUCTION

Hip dysplasia typically manifests itself in adolescence or young adulthood, after decades of subclinical development. Symptomatic hip dysplasia is shown to reduce physical activity as walking and increase self-reported pain scores. Hip dysplasia significantly increase the risk of developing secondary hip osteoarthrosis, why this population is offered periacetabular osteotomy (PAO), as PAO outcome studies have demonstrated major clinically important improvements in pain, function, quality of life and activity level.

In spite of careful attention to perioperative pain management after PAO pain and nausea remain a major challenge to the patients. The sizable need for opioids increase the risk of opioid-related side effects in a low-comorbidity population already in increased risk of PONV. Therefore, it is relevant to investigate regimens to reduce opioid consumption and PONV.

To our knowledge, no experience with glucocorticoids as analgesic in PAO patients exists. As the PAO-procedure leads to major surgical trauma and pain, we consider it likely that these patients will benefit from an added glucocorticoid administration, based on the assumption that perioperative glucocorticoid treatment in other orthopaedic procedures with major trauma has shown significant reduction in pain and PONV.

A regime including a repeated postoperative glucocorticoid administration is expected to result in a clinically relevant reduction in opioid consumption and opioid-related side effects. Generally, analgesic regimes including administration of repeated glucocorticoid doses are not sufficiently investigated.

### Objectives

The primary objective of this trial is to compare the effect of intravenous dexamethasone (DXMT independent of dose), relative to placebo, on cumulated postoperative morphine consumption in milligrams during 48 hours from baseline. Morphine administered both as patient-controlled analgesia (PCA), i.v. morphine and any other supplemental morphine administered postoperatively. Unit of analysis for this consumption will be evaluated as a single figure measured in milligram (mg).

The primary null hypothesis is that there is no difference between groups regarding morphine consumption after PAO (i.e., $H_0$: $\mu_{Dexa} = \mu_{Placebo}$; meaning that the primary analyses will be performed independent of the dose of Dexamethasone [once and twice daily combined]).

Key secondary objectives will be to compare the effect of intravenous dexamethasone (combined dose DXMT), relative to placebo, on the following outcome measures:

- Postoperative pain intensity during rest at 24 hours. Pain intensity is assessed using the visual analogue scale (VAS) (0-100mm).
- Postoperative pain intensity during activity at 24 hours. Pain is evaluated under the timed-up-and-go (TUG) procedure. Pain intensity is assessed using the visual analogue scale (VAS) (0-100mm).
- Postoperative pain intensity during rest at 48 hours. Pain intensity is assessed using the visual analogue scale (VAS) (0-100mm).
- Postoperative pain intensity during activity at 48 hours. Pain is evaluated under the timed-up-and-go (TUG) procedure. Pain intensity is assessed using the visual analogue scale (VAS) (0-100mm).

- Cumulated postoperative morphine consumption from 48 hours until day 14 post operation [Time Frame: 48 hours - day 14 postoperatively] Morphine consumption in milligrams after the operation.

- Postoperative nausea and vomiting [ Time Frame: 24 hours postoperatively ] Nausea and vomiting are evaluated using a 4 point scale: none, mild, moderate, severe

- Postoperative nausea and vomiting [ Time Frame: 48 hours postoperatively ] Nausea and vomiting are evaluated using a 4 point scale: none, mild, moderate, severe

- Antiemetic consumption [Time Frame: 0-48 hours postoperatively] Evaluation of cumulated antiemetic consumption in mg and drug will be assessed.

- Timed up and go test [Time Frame: 24 hours postoperatively] Is assessed from; the time that the participant takes to rise from a chair, walk three meters, turn around, walk back to the chair, and sit down.

- Timed up and go test [Time Frame: 48 hours postoperatively] Is assessed from; the time that the participant takes to rise from a chair, walk three meters, turn around, walk back to the chair, and sit down.

Secondary objectives will be outlined to include comparison of the three intervention groups in different setups: If statistically significant difference between placebo and the combined groups of DXMT is found, the two DXMT groups will be compared afterwards.

Deviation from ClinicalTrials.gov registration: "Sleep quality assessed using the VAS scale" was registered as a secondary outcome on ClinicalTrials.gov, but data on this outcome was intentionally not collected as considered less important without sleep monitoring. This was decided before study initiation and corrected in the study protocol, but by mistake not deleted on ClinicalTrials.gov.

## STUDY METHODS

### Trial design

The PAODEX trial is a single-centre, randomised (allocation 1:1:1), parallel-group, placebo-controlled, superiority trial with three parallel groups. Patients are recruited from the orthopaedic department at Odense University Hospital (OUH) in the Region of Southern Denmark. Participants signing the informed consent were randomised to one of three groups:

1. Placebo at induction of anaesthesia and placebo (isotonic saline) 24 hours after surgery (PLB)
2. DXMT at induction of anaesthesia and placebo 24 hours after surgery (Single D)
3. DXMT at induction of anaesthesia and DXMT 24 hours after surgery (Double D)

The treatments are described in detail in the trial protocol.

The statistical analysis plan (SAP) and trial protocol are reported in accordance with the *"Guidelines for the Content of Statistical Analysis Plans in Clinical Trials"* and *"Standard Protocol Items: Recommendations for Interventional Trials"* (SPIRIT), respectively, while reporting of the trial will follow the *"Consolidated Standards of Reporting Trials"* (CONSORT) statement.

Trial registration was performed at ClinicalTrials.gov (NCT03874936) in March 2019. Patient enrolment started in November 2020. The trial has been approved by The Regional Committees on Health Research Ethics for Southern Denmark (Project-ID S-20190017) and The Danish Data Protection Agency (Journal No 19/10040).

**Randomisation**

Patients were randomised with a 1:1:1 allocation as per a computer-generated randomisation schedule. An independent data manager developed the computer-generated list of random numbers using the randomisation tool in Research Electronic Data Capture (REDCap). Administrators of the randomisation procedure were blinded to randomisation at all times during the trial. The randomisation code is stored in REDCap with no access form the author group.

**Sample size and power calculation**

In the planning of the PAODEX Trial a small pilot study (10 patients) was launched for the clinicians to have some empirical data to support their clinical hypothesis (e.g., practical for power and sample size considerations). Two patients were exposed to perioperative DXMT administration (participants #9 and #10), whereas data was collected "as usual" from 8 patients (#1-#8; corresponding to placebo *per se*). Based on the sparse data available from the pilot study, basic descriptive statistics indicate that the overall mean is 47.47 mg morphine (over the 48 hours primary interest), with a standard deviation (SD) of 32.69 mg morphine; these descriptive statistics together with the overall median (57.9 mg morphine) suggests that data might not be normally distributed, however. Although questionable - because of the small sample sizes - the mean (SD) in the placebo and DXMT group corresponded to 56.8 (32.2) and 20.0 (14.1) mg morphine, respectively. We note that the poorly controlled – but potential clinical effect of adding DXMT – could correspond to an estimated treatment difference of promising 36.8 mg morphine *per se* (i.e., potential net benefit).

For a two-sample pooled t test of a normal mean difference with a two-sided significance level of 0.05, assuming a common standard deviation of 40 mg morphine, a total sample size of 87 patients assuming an allocation ratio of 2 to 1 (corresponding to group A&B vs group C) is required to obtain a power of at least 90% to detect an estimated treatment difference of 30 mg morphine. It was decided to aim for enrolment of 90 participants in total (randomised: 30:30:30; i.e., $n_A + n_B = 60$ vs $n_C = 30$).

Based on the same assumptions as above, even if we are only able to enrol and randomise 66 patients in total (allocation ratio of 2 to 1; i.e., approximately 44 vs 22 patients) we will obtain a reasonable statistical power of 80%.

According to our key secondary efficacy objectives we also want to compare the effect of double dose (Group A) with a single dose (Group B) of intravenous DXMT: For a two-sample pooled t test of a normal mean difference with a two-sided significance level of 0.05, assuming a common standard deviation of 40 mg morphine, a total sample size of 60 patients assuming a balanced design ($n_A = n_B = 30$) has a statistical power of 81.5% to detect an estimated treatment difference of 30 mg morphine (still assuming that the SD is around 40 mg).

### Timing of final analysis

The final analysis for the between-group comparison (placebo versus DXMT) for the primary endpoint is planned to be performed after each randomised patient has completed 8 weeks follow-up. The main trial publication will be prepared when these data have been received and cleaned (anticipated by April 2023).

In subsequent manuscripts, secondary long-term endpoints will be analysed when the 12 months (anticipated January 2024) have been reached for all randomised patients followed by preparation of manuscripts.

## STATISTICAL PRINCIPLES

### Analysis populations

The primary analyses will be based on the intention-to-treat (ITT) population. Thus, all data from individuals randomised regardless of dropout and participation rate will be included in the ITT analysis belonging to the initially allocated. In general, it is advantageous to demonstrate a lack of sensitivity of the principal trial results to alternatives of the set of participants analysed (i.e., other analysis populations).

For the main analysis, we will conduct analyses of the full analysis set (according to the ITT population, using appropriate statistical techniques to handle missingness), and two subsequent sensitivity analyses (A) a per protocol analysis, as well as (B) a non-responder imputation so that any differences between them can be explicitly discussed and interpreted. The per protocol population will be defined as participants who were (*i*) randomised, AND (*ii*) had the intended prescription (randomly allocated intervention), AND (*iii*) had a successfully measured primary endpoint.

### Statistical analyses and multiplicity considerations

The primary null hypothesis is based on the comparison between participants randomised to any dose of DEXA (group A&B) vs C; $H_0$: $\mu_{A+B} = \mu_C$.

The overall study design is based on three groups (A, B, and C); we apply linear modelling based on general linear models (GLMs) in the analysis of the primary and key secondary outcome measures (all continuous outcome measures): Analysis of covariance (ANCOVA) models will be performed, which produces several diagnostic measures, provides contrasts and estimates for customised hypothesis tests, and it provides tests for means adjusted for covariates (e.g., least squares [LS] means adjusted for baseline variables). The GLM procedure handles models relating a continuous dependent variable to one or several independent variables. The main statistical estimand from these analyses will be the difference between the least squares mean reported with 95% Confidence Intervals (95%CIs). Categorical outcomes related to dichotomous endpoints will be analysed with the use of logistic regression with the same fixed effects and covariates as the respective analysis of covariance; for ease of interpretation the resulting Odds Ratios (95%CI) will subsequently be converted into Risk Differences with 95%CIs.

The independent variables can be either classification variables, which divide the observations into discrete groups (e.g., A&B and C, respectively), or continuous variables (e.g., the level at baseline). As stated above, we will analyse continuous outcomes using ANCOVA models, with a fixed factor for group (either 2

levels for primary models [A&B pooled vs C] or 3 levels for the secondary analyses [A vs B vs C], respectively) and adjust for the value at baseline.

For the secondary objectives comparing all three groups, each continuous outcome variable (after performing the overall ANCOVA model), obtain the *P*-value and group contrast for the difference between the LS means: A vs. B (in analogy to a two-sample t test), independent of what the overall ANCOVA model indicates. Also, results following the ANCOVA model will be expressed as estimates of the group difference in the various pairwise comparisons in the changes from baseline, with 95% CIs to represent the precision of the estimates.

To assess the adequacy of the linear models describing the observed data, as well as to check the assumptions for the systematic and random parts of the models, we will investigate the model features via the predicted values and the residuals; that is, the residuals have to be normally distributed (around 0) and be independent of the predicted values.

All reported *P*-values and 95%CIs will be two-sided and will not be adjusted for multiple comparisons per default (despite having 4 potential tests for each outcome variable $[3 \times \{3 - 1\}/2]$ plus the overall ANCOVA). Per default, we set the statistical significance at the conventional level of 0.05 ($P < 0.05$), but the findings will be interpreted cautiously based on the proposed hierarchy presented in this SAP. All analyses will be performed using commercially available statistical software (i.e., SAS studio). The existing 'Statistical Analysis Plan' (SAP) was finalised prior to the commencement of any data analysis.

**TRIAL POPULATION**

**Screening data**

The total number of patients screened for eligibility will be collected and presented in a CONSORT flowchart to describe representativeness of the trial sample (Figure 1). Furthermore, the number of ineligible patients randomised by mistake, if any, will be reported including reason for ineligibility.

**Eligibility**

Patients conforming to following inclusion and exclusion criteria are considered eligible for the trial.

*INCLUSION CRITERIA:*

1. Patients undergoing PAO due to symptomatic hip dysplasia (CE<25grader) or retroverted acetabulum (crossover and posterior wall sign)
2. $\geq$ 18 years
3. Females if fertile*: Verified negative s-HCG, usage of safe contraceptives** or surgical sterilisation.
4. Patients who give their written informed consent to participating in the trial, after having fully understood the content of the protocol and restrictions.
   *Postmenopausal is defined as >12months amenorrhoea.
   **Safe contraceptives accepted: intrauterine device or hormone contraceptives.

*EXCLUSION CRITERIA:*

1. Patients who cannot speak or understand Danish

2. Allergy or contraindications to trial medication
3. Spinal anaesthesia
4. Second intervention carried out simultaneously (e.g. femur osteotomy)
5. Patients with daily opioid consumption prior to surgery (tramadol and codeine accepted)
6. Drug, medical abuse or weekly alcohol consumption beyond ≥7 (female) and ≥14 (men) units, respectively.
7. Mental disability, anxiety disorder (active psychiatric disorder or consumption of tricyclic antidepressants)
8. Diabetes diagnosed prior to inclusion
9. Immune suppression therapy (e.g. systemic glucocorticoids)
10. Kidney impairment (eGFR < 50ml/min) or liver disease (≥Child Pugh B)

**Recruitment**

The CONSORT flowchart will comprise number of patients screened, excluded (with reasons) eligible for inclusion in the trial, randomised, receiving their allocated treatment, withdrawals (with reasons), and lost to follow-up (with reasons), included in the ITT analysis, included in per protocol analysis, and included in as treated analysis. The CONSORT flowchart is depicted in **Figure 1**.

**Withdrawal / follow-up**

The level of consent withdrawal will be classified as the following two options: 1) consent to continue follow-up and data collection and 2) complete withdrawal with no further follow-up and data collection. Timing of withdrawal and loss to follow-up will be presented in the CONSORT flowchart with numbers and reasons for withdrawal and/or loss to follow-up. Furthermore, the number (with reasons) of loss to follow-up during the course of the trial will be summarised by treatment group.

**Missing data and sensitivity analyses**

Statistical methods to handle missing data: Loss to follow-up (and missing data for various reasons) is hard to avoid in randomised trials. We will apply the analysis framework suggested by White et al for "ITT analyses" that depends on making plausible assumptions about the missing data and including all participants in sensitivity analyses:

1. Attempt to follow up all randomised participants, even if they withdraw from allocated treatment

2. Perform a main analysis of all observed data that are valid under a plausible assumption about the missing data (i.e., 'Missing At Random' [MAR], applying multiple imputation to handle missing outcome data)

3. Perform sensitivity analyses to explore the effect of departures from the assumption made in the main analysis (i.e., using both the Per Protocol population as well as the simplistic non-responder imputation).

4. Account for all randomised participants, at least in the sensitivity analyses.

The evaluability assessment of each participant in the statistical analyses will be performed before the code is revealed. Excluded participants and missing, unused or false data will be described.

Protocol deviations will be reported (defined as a failure to adhere to the protocol such as the wrong intervention being administered, incorrect data being collected and documented, errors in applying inclusion/exclusion criteria or missed follow-up visits). These will be defined as major or minor.

Protocol deviations will be defined prior to unblinding of data to avoid any bias being caused and due consideration given to inclusion of participants within analysis populations.

Explicit data analysis aspect (incl. computational syntax) and the observed protocol deviations may be reported in another document - the Statistical Analysis Plan (SAP); the SAP was written and confirmed with closure as a pdf with date prior to unblinding of data to avoid any bias being caused and due consideration given to inclusion of participants within analysis populations.

**FIGURE 1**

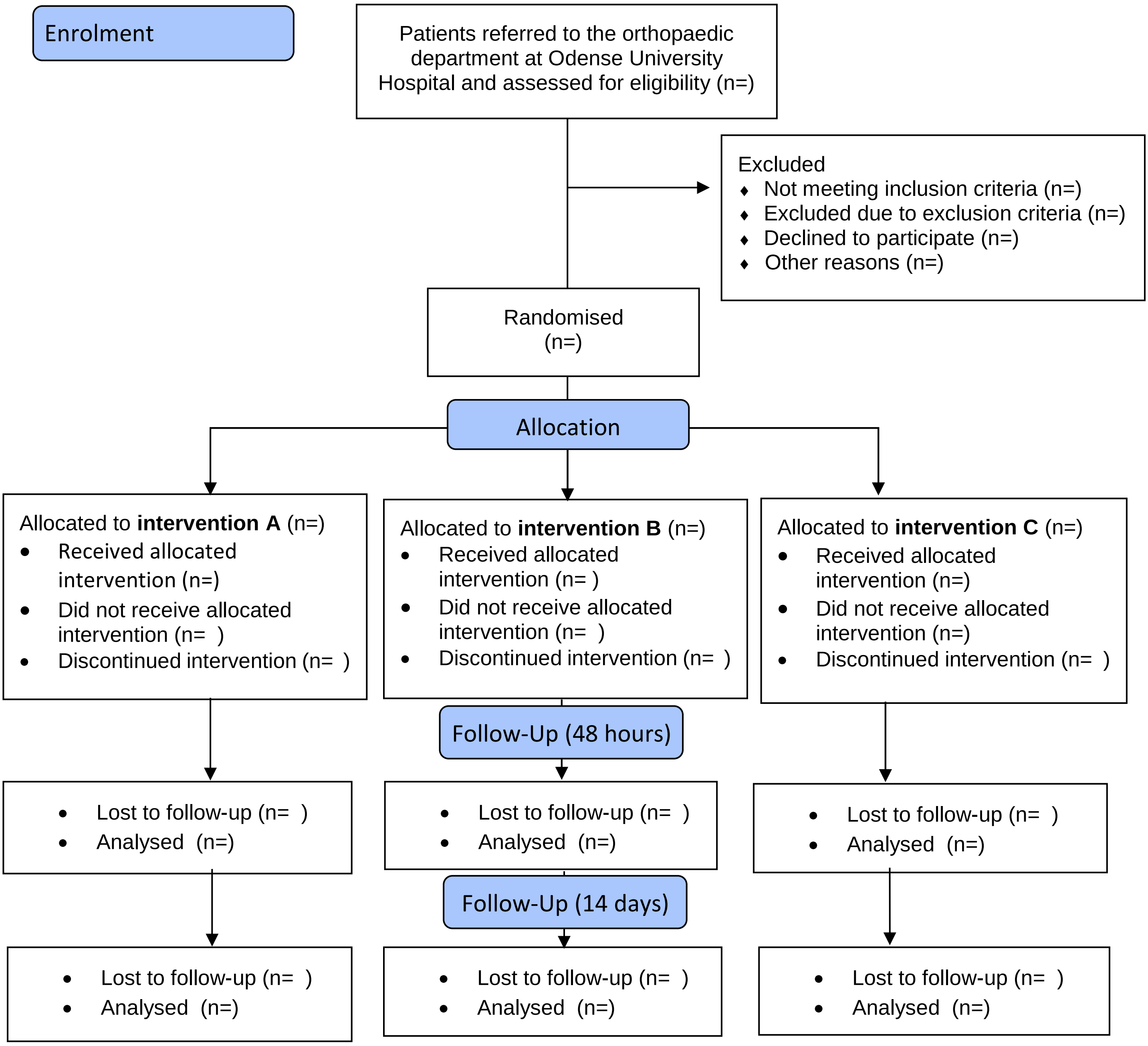


**Figure 1.** CONSORT flowchart.

**Table 1. Demographic and Clinical Characteristics of the Patients at Baseline in the ITT Population**

| Characteristic | Placebo group (N=??) | DXMT groups (N=??) | Combined (Total N=??) |
|---|---|---|---|
| Female sex - no. (%) | | | |
| Age - yr | | | |
| Height – m | | | |
| Weight – kg | | | |
| Body Mass Index – kg/m$^2$ | | | |
| Score on the ASA physical-status classification: | | | |
| Class I, no. (%) | | | |
| Class II, no. (%) | | | |
| Class III, no. (%) | | | |
| Substance use: | | | |
| Current smoker, no. (%) | | | |
| Alcohol: | | | |
| 0 units/week, no. (%) | | | |
| 1-7 units/week, no (%) | | | |
| 8-14 units/week, no (%) | | | |
| >14 units/week, no (%) | | | |
| Side of surgery - dxt, no. (%) | | | |
| Duration of surgery - min | | | |
| Fluid administration - mL | | | |
| Bleeding - mL | | | |
| Propofol, cumulated dose - mg | | | |
| Remifentanil, cumulated dose - mg | | | |
| Fentanyl, cumulated dose - µg | | | |
| VAS, awakening ward (points 0 - 100) | | | |

* Will be reported as Plus–minus values for means SD, unless otherwise stated. Percentages may not sum to 100 because of rounding.

**Table 2. Primary and Ten Key Secondary Outcomes in the ITT Population**

| Characteristic | Placebo group (N=??) | DXMT groups (N=??) | Difference (95% CI) | *P*-value |
|---|---|---|---|---|
| Cumulated postoperative morphine consumption, up to 48 hours - mg | | | | |
| *Postoperative pain intensity after 24 hours - VAS, at rest (points 0 - 100) | | | | |
| *Postoperative pain intensity after 24 hours - VAS, during activity (points 0 - 100) | | | | |
| *Postoperative pain intensity after 48 hours - VAS, at rest (points 0 - 100) | | | | |
| *Postoperative pain intensity after 48 hours - VAS, during activity (points 0 - 100) | | | | |
| *Cumulated postop. morphine from 48 hrs to 14 days - mg | | | | |
| *Postop. nausea and vomiting at 24 hrs – (points 0 – 3) | | | | |
| *Postop. nausea and vomiting at 48 hrs - (points 0 – 3) | | | | |
| *Postop. antiemetic consumption at 48 hrs – cumulated mg | | | | |
| *Timed up and go test 24 hours - sec | | | | |
| *Timed up and go test 48 hours - sec | | | | |

* Will be reported for each group as Least Squares Means with Standard Errors while the Difference between them will be reported with 95% Confidence intervals. Continuous endpoints will be analyzed using an analysis of covariance (ANCOVA) model with randomized treatment, stratification groups, and baseline outcome value as covariate. Missing data will be imputed from all retrieved patients and the results will be combined using Rubin's rules. Categorical endpoints will be analyzed by logistical regression.

**Table 3. Reporting of Harms**

| Characteristic | Placebo group | DXMT/placebo | DXMT/DXMT |
|---|---|---|---|
| Serious adverse events (SAE), up to 8 weeks after operation, no. (%) | | | |
| Deep infection, no. (%) | | | |
| Revision due to acetabular/femoral fracture, no. (%) | | | |
| Deep venous thrombosis/pulmonary embolism, no. (%) | | | |
| Nerve injury, no. (%) | | | |
| Death, no. (%) | | | |

* Will be reported as numbers and percentages (no. [%])

**Table 4. Optional Exploratory Analyses Primary and Ten Key Secondary Outcomes in the ITT Population**

| Characteristic | Placebo (N=??) | DXMT/placebo (N=??) | DXMT/DXMT (N=??) | Difference: Double vs Single Dose (95% CI) |
|---|---|---|---|---|
| Cumulated postoperative morphine consumption, up to 48 hours - mg | | | | |
| *Postoperative pain intensity after 24 hours - VAS, at rest (points 0 - 100) | | | | |
| *Postoperative pain intensity after 24 hours - VAS, during activity (points 0 - 100) | | | | |
| *Postoperative pain intensity after 48 hours - VAS, at rest (points 0 - 100) | | | | |
| *Postoperative pain intensity after 48 hours - VAS, during activity (points 0 - 100) | | | | |
| *Cumulated postop. morphine from 48 hrs to 14 days - mg | | | | |
| *Postop. nausea and vomiting at 24 hrs - (points 0 – 3) | | | | |
| *Postop. nausea and vomiting at 48 hrs - (points 0 – 3) | | | | |
| *Postop. antiemetic consumption at 48 hrs – cumulated mg | | | | |
| *Timed up and go test 24 hours - sec | | | | |
| *Timed up and go test 48 hours - sec | | | | |

* Will be reported for each group as Least Squares Means with Standard Errors while the Difference between them will be reported with 95% Confidence intervals. Continuous endpoints will be analyzed using an analysis of covariance (ANCOVA) model with randomized treatment, stratification groups, and baseline outcome value as covariate. Missing data will be imputed from all retrieved patients and the results will be combined using Rubin's rules. Categorical endpoints will be analyzed by logistical regression (reported as an Odds Ratio with 95%confidence interval).

**Appendix Table 1. Sensitivity Analysis Table, Primary and Ten Key Secondary Outcomes in the Per Protocol Population**

| Characteristic | Placebo group (N=??) | DXMT groups (N=??) | Difference (95% CI) |
|---|---|---|---|
| Cumulated postoperative morphine consumption, up to 48 hours - mg | | | |
| *Postoperative pain intensity after 24 hours - VAS, at rest (points 0 - 100) | | | |
| *Postoperative pain intensity after 24 hours - VAS, during activity (points 0 - 100) | | | |
| *Postoperative pain intensity after 48 hours - VAS, at rest (points 0 - 100) | | | |
| Postoperative pain intensity after 48 hours - VAS, during activity (points 0 - 100) | | | |
| *Cumulated postop. morphine from 48 hrs to 14 days - mg | | | |
| *Postop. nausea and vomiting at 24 hrs - (points 0 – 3) | | | |
| *Postop. nausea and vomiting at 48 hrs - (points 0 – 3) | | | |
| *Postop. antiemetic consumption at 48 hrs – cumulated mg | | | |
| *Timed up and go test 24 hours - sec | | | |
| *Timed up and go test 48 hours - sec | | | |

The per protocol population will be defined as participants who were (i) randomized, AND (ii) had the intended prescription (randomly allocated intervention), AND (iii) had a successfully measured primary endpoint. Will be reported for each group as Least Squares Means with Standard Errors while the Difference between them will be reported with 95% Confidence intervals. Continuous endpoints will be analyzed using an analysis of covariance (ANCOVA) model with randomized treatment, stratification groups, and baseline outcome value as covariate. Missing data will be imputed from all retrieved patients and the results will be combined using Rubin's rules. Categorical endpoints will be analyzed by logistical regression (reported as an Odds Ratio with 95%confidence interval).

**Appendix Table 2. Sensitivity Analysis Table, Primary and Ten Key Secondary Outcomes in the Non-Responder Population**

| Characteristic | Placebo group (N=??) | DXMT groups (N=??) | Difference (95% CI) |
|---|---|---|---|
| Cumulated postoperative morphine consumption, up to 48 hours - mg | | | |
| *Postoperative pain intensity after 24 hours - VAS, at rest (points 0 - 100) | | | |
| *Postoperative pain intensity after 24 hours - VAS, during activity (points 0 - 100) | | | |
| *Postoperative pain intensity after 48 hours - VAS, at rest (points 0 - 100) | | | |
| *Postoperative pain intensity after 48 hours - VAS, during activity (points 0 - 100) | | | |
| *Cumulated postop. morphine from 48 hrs to 14 days - mg | | | |
| *Postop. nausea and vomiting at 24 hrs - (points 0 – 3) | | | |
| *Postop. nausea and vomiting at 48 hrs - (points 0 – 3) | | | |
| *Postop. antiemetic consumption at 48 hrs – cumulated mg | | | |
| *Timed up and go test 24 hours - sec | | | |
| *Timed up and go test 48 hours - sec | | | |

Will be reported for each group as Least Squares Means with Standard Errors while the Difference between them will be reported with 95% Confidence intervals. Continuous endpoints will be analyzed using an analysis of covariance (ANCOVA) model with randomized treatment, stratification groups, and baseline outcome value as covariate. Missing data will be imputed from all retrieved patients and the results will be combined using Rubin's rules. Categorical endpoints will be analyzed by logistical regression (reported as an Odds Ratio with 95%confidence interval).
